# Designing Natural Polymer-Based Capsules and Spheres for Biomedical Applications—A Review

**DOI:** 10.3390/polym13244307

**Published:** 2021-12-09

**Authors:** Kusha Sharma, Ze’ev Porat, Aharon Gedanken

**Affiliations:** 1 Department of Chemistry, Bar-Ilan Institute for Nanotechnology and Advanced Materials, Bar-Ilan University, Ramat-Gan 52900, Israel; kusha28793@gmail.com; 2 Department of Civil and Environmental Engineering, Ben-Gurion University of the Negev, Be’er Sheva 84105, Israel; 3 Department of Chemistry, Nuclear Research Center-Negev, Be’er Sheva 84190, Israel

**Keywords:** natural polymers, polymeric capsules, porous polymeric spheres, active pharmaceutical carriers, drug delivery, stimuli-responsive release, cell culture platforms

## Abstract

Natural polymers, such as polysaccharides and polypeptides, are potential candidates to serve as carriers of biomedical cargo. Natural polymer-based carriers, having a core–shell structural configuration, offer ample scope for introducing multifunctional capabilities and enable the simultaneous encapsulation of cargo materials of different physical and chemical properties for their targeted delivery and sustained and stimuli-responsive release. On the other hand, carriers with a porous matrix structure offer larger surface area and lower density, in order to serve as potential platforms for cell culture and tissue regeneration. This review explores the designing of micro- and nano-metric core–shell capsules and porous spheres, based on various functions. Synthesis approaches, mechanisms of formation, general- and function-specific characteristics, challenges, and future perspectives are discussed. Recent advances in protein-based carriers with a porous matrix structure and different core–shell configurations are also presented in detail.

## 1. Introduction

Conventional drug therapy involves administering the drug or pharmaceutical agent directly into the body, through oral, pulmonary, or parenteral routes. However, several demerits to this approach are the rapid release of the drug into the body at the site of administration, loss of drug dose on the way from the site of administration to the target site (due to biological degradation), the requirement for administering higher doses of the drugs to compensate for this loss, higher chances of over- or under-medication, side effects due to the interaction of the drugs with untargeted sites, the requirement of frequent dosing, lower drug bioavailability, lower per-unit cost (but higher overall healthcare cost), and higher total dosage requirement into the body. These demerits have led to the need for a different approach, which involves transporting the active pharmaceutical cargo (APC) and releasing it to the targeted (or affected) site in the body for therapeutic effect via drug delivery agents. Such a therapeutical approach has enabled the site-specific, slow, sustained, and controlled release of drugs, thus improving their bioavailability, pharmacokinetics, and increased efficacy, as well as minimizing the side effects to the untargeted sites and overall risk to the patient, thereby reducing the overall medication cost, due to the decreased frequency of drug administration and increasing patient compliance. 

The development of drug delivery systems (DDS) began in the 1950s, when Jatzkewitz et al. (1955) reported that the conjugation of the psychedelic drug Mescaline, with co-polymer of N-vinylpyrrolidone and acrylic acid, prolonged its in-vivo residence time [1]. The first generation of drug delivery (1950–1980) involved the study of controlled-release mechanisms and development of oral and transdermal sustained-release systems [2]. Eventually, the first controlled delivery device, based on silicone rubber for delivering the drug isoproterenol, was reported in 1964 for its potential application as implants to treat heart block [3]. This was followed by several studies on developing a variety of polymeric and liposomal systems for the controlled release of various drugs and their underlaying release mechanisms [4,5,6,7]. The second-generation drug delivery (1981–2010) was basically focused on the study and development of constant-release, self-regulated drug delivery systems, and nanoparticle-based drug delivery systems. During this era, many sustained-release drug formulations (drugs-DDS), based on polymeric nanoparticles (Adagen, Gliadel, Copaxone), polymeric implants (Zoladex), liposomal carriers (Doxil, Abelcet), dendrimer-conjugates, and protein-based nanoparticles (Abraxane), were clinically tested and approved by the FDA. The past decade has been focused on designing smart, stimuli-responsive systems for targeted drug delivery. These systems have been shown to actively deliver the drug to the target site and enable controlled drug release by undergoing physical and/or chemical changes, in response to biological or external triggers. 

**Figure 1:**
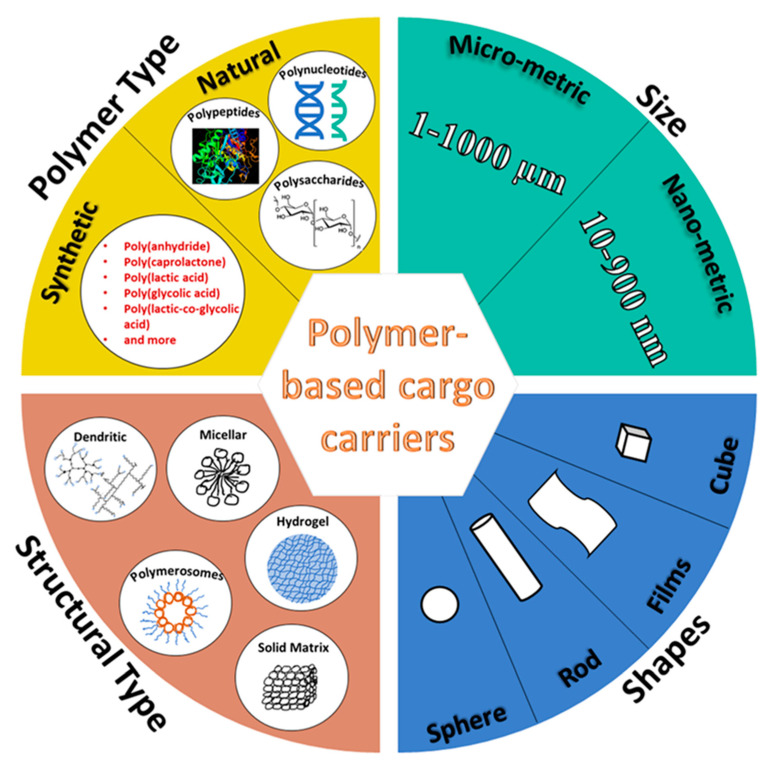
Classification of polymer-based carriers of biomedical cargo.

In the past few decades, a wide variety of novel drug delivery approaches, in the form of micro- and nanoparticles (core–shell capsules, as well as matrix-type spheres), transdermal patches, gels, dendrimers, micelles, microneedles, and microfluidics-based devices have been developed (Figure 1). These were usually made of synthetic polymers (such as poly-lactic glycolic acid), natural polymers (such as polysaccharides, polypeptides, and polynucleotides) (see Table 1), liposomes, metallic formulations, metal oxides, carbon nanotubes, etc., aimed at a variety of functions, including site-selective, active, or passive targeted delivery of a wide variety of drugs for treating diseases, such as cancer and diabetes. Several parameters, such as the material of fabrication, size, shape, structural configuration, and surface characteristics of these APC carrier systems (ACSs), play a major role in their interaction with the in-vivo chemical environment, while passing, from the site of administration to the site of action, their function and in-vivo biodistribution. As such, these parameters are considered vital to designing better and smarter ACSs.

The following review focuses on function-specific aspects of designing micro- and nano-metric spherical APC carrier systems (SACS) made of natural polymers, such as polysaccharides and polypeptides, having structural configurations of core–shell and porous matrix. Chemical aspects involved in designing SACS, their synthesis approaches, formation mechanisms, and general- and application-specific characteristics are discussed. Finally, recent advances in the protein-based SACS, with a porous matrix structure, as well as different core–shell configurations, are presented in detail.

**Table 1:** List of natural polymers utilized to develop biomedical carriers.

Polymer Class	Polymer
Polysaccharides	Cellulose
	Cellulose derivatives
	Alginate
	Gellan gum
	Pectin
	Gum Arabica
	Gaur gum
	Locust bean gum
	Starch
	Carrageenan
	Chitin
	Chitosan
	Xanthan gum
	Shellac
	Dextran
	Cashew gum
	Pullulan
Polypeptides	Gelatin
	Bovine serum albumin
	Human serum albumin
	Egg albumin
	Casein
	Collagen
	Keratin
	Elastin
	Resilin
	Soy protein
	Gliadin
Hyaluronic acid	Hyaluronic acid
Phospholipids	Liposomes
Polynucleotides	Ribonucleic acid
	Deoxyribonucleic acid

## 2. Chemical Aspects of Designing Natural Polymer-Based Spherical Capsules and Spheres

Designing nano- and micro-capsules calls for the foremost consideration of the requirements laid down for their utilization in various biomedical functions. Such functions may involve sustained-release of cargo at the affected site in the body [8], the stimuli-responsive release of the cargo [9], its targeted delivery to the site of action [10], its protection from the hostile bodily environment [11], its better bioavailability in the body [12], better integration of the cargo into the body (such as the integration of the progenitor cells at tissue lesions) [13], blood vessel embolization [14] by the capsules, etc. The structural configurations of the SACS and mode of encapsulation of the cargo are chosen based on these biomedical requirements, upon which the eventual path/technique of capsule synthesis depends. Here, we discuss these requirements in detail, their decisive role in structural configuration choice and parameters, and the synthesis approaches that have been employed for years in the development of natural polymer-based SACS.

### 2.1. Function-Specific Carrier Design

#### 2.1.1. Structural Configurations and Carrier Materials

**Figure 2:**
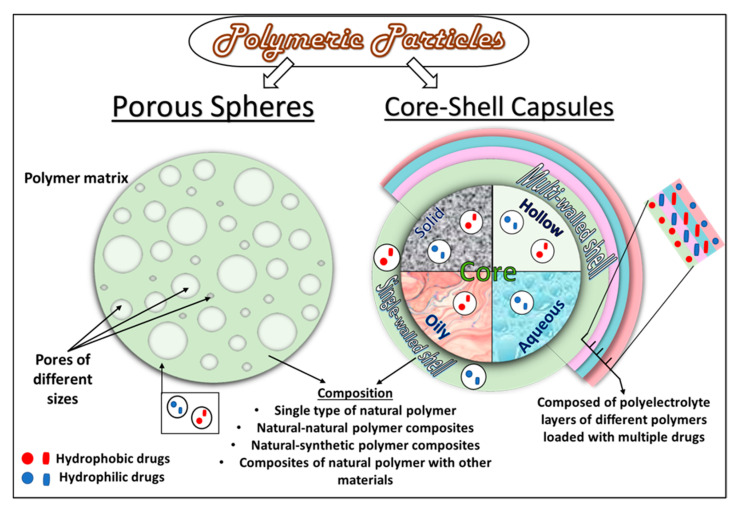
Structural configurations of core–shell and porous natural polymeric/protein particles.

Through the years of evolution in micro- and nano-capsule design, several core–shell configurations have been developed. These include core–shell capsules with solid, liquid, or hollow cores, encapsulated within a single or multiwalled shell and made of natural polymers, such as carbohydrates and proteins. Porous spherical matrices have also been prepared as cargo carriers. It is important to note that the polymer-based, micro- and nano-metric core–shell capsules and porous spheres fall under a broader category of micro- and nano-particles, with sizes ranging from 1–1000 µm and 10–900 nm, respectively. A general schematic diagram of the different structural configurations is presented in Figure 2.

Polymeric capsule shells, with a variety of compositions, have been prepared. These shell compositions may involve (a) a single type of natural polymer, such as chitosan [15] or albumin [16]; (b) composites of different types of natural polymers, such as BSA-alginate [17]; (c) composites of different types of natural and synthetic polymers, such as collagen-PLGA [18]; (d) natural polymers functionalized by other materials, including inorganic nanoparticles [19], functionalizing polymers [10], antibodies [20], and a variety of other materials. Diverse core materials have been encapsulated, in solid [21] or liquid form [22,23], within these shells, either as carriers of different types of active pharmaceutical cargo (APC) or made directly of the solid or liquid APCs (see Figure 2). A solid core of various natural [11] as well as synthetic polymers [24], metallic particles [23], and composites, have been prepared to make up a hydrophilic or a hydrophobic core, depending upon the type of moieties present in the precursor materials and application-based requirements. Liquid cores of organic solvent [22], oils [16], and a variety of aqueous media [25] have also been prepared to disperse either hydrophobic or hydrophilic APCs. In addition, hollow/porous capsules, made of natural polymers, have also been developed [18,26]. Depending upon the biomedical applications, the cargo may be dispersed or dissolved in the liquid/solid core as a reservoir/matrix or/and embedded in the shell of the capsules with a liquid/solid/hollow core. Depending upon the desired applications, the encapsulated cargo can be medicinal drugs [10], growth factors [12], stem cells, progenitor cells [27], probiotic bacterial strains [11], nutritional molecules (such as vitamins [28]), hormones (such as insulin [29]), and several more.

##### Desired Functions

*Sustained-release.* The choice of the core–shell materials depends primarily upon the physical and chemical properties of the natural polymer, type of applications, and mode of action required. Sustained-release formulations are prepared from the natural polymeric shell and core materials that facilitate the prolonged-release of APC via a combination of processes, such as diffusion, erosion, osmosis, and swelling. These processes are discussed in detail in the next section. Purely diffusion-controlled release from a capsule primarily involves the mass transfer of the cargo from the capsule to the release media, driven solely by their concentration gradient [30]. However, generally, release capsules made of natural polymers undergo a combination of dissolution, swelling, and erosion processes to release the cargo at the target site. Silk fibroin-based microcapsules have shown swelling-controlled release of doxorubicin (Dox) [14], wherein the microcapsules experienced enormous water uptake, leading to the enhanced initial release of Dox, and eventually swelled-up, due to which the Dox release rate slowed down. Collagen microcapsules have shown erosion-controlled release of human vascular endothelial growth factor (rhVEGF) and basic fibroblast growth factor (bFGF) over time [28,31].

*Stimuli-responsive release.* In addition, the cargo release processes may also be triggered in response to certain stimuli, such as a change in pH and temperature or the presence of digestive enzymes. Ionic polysaccharide-based capsules of chitosan, alginate, agar, carrageenan, cellulose, gaur, and xanthan gum have shown pH- and temperature-responsive release, due to the sensitivity of certain groups (such as amine group in chitosan) towards certain pH and higher temperature. Similarly, pectin and chondroitin sulfate show pH sensitivity and enzymatic degradation [32]. Various proteins, such as albumins, show pH, temperature, and enzyme responsive release of cargo. The pH-responsive action has also been shown in polymer–polymer composite microcapsules of BSA-alginate [17]. Additionally, the enzyme-catalyzed release of 3,4,9,10-tetra-(hectoxy-carbonyl)-perylene (THCP) was observed from BSA/polyphenol microcapsules, due to their degradation by α-chymotrypsin [33]. Cargo release, in the cases above, may involve both the release from the capsule core, as well as the capsule shell, depending upon the location and the state of the APC in the capsule. 

*Targeting.* Targeted delivery of cargo refers to delivering an APC to the target site, selectively and independently of the route (site and method) of administration, through a delivery agent. Targeting can be organ-specific, tissue-specific, specific to pathogens (such as parasites), receptor-specific, or specific at the organelle-level for targeting mitochondria, cytoplasm, DNA, etc. A higher concentration of the drug at the desired site can be ensured through targeted delivery by preventing undesired drug loss and adverse effects at the untargeted sites. For targeted delivery of APC, the capsule shell is usually functionalized with various ligands, such as peptides [34], polymers [35], antibodies [20], nucleic acids, and vitamins [22]. Physically stimulated targeted delivery formulations have also been developed, wherein superparamagnetic particles have been functionalized on the microcapsule shell for the magnetically stimulated delivery of capsules at the target site [19]. 

*Protection of the cargo.* Another function of the capsules involves the protection of the cargo from the hostile bodily environment. Cargo, such as hydrophilic drugs and probiotic bacterial strains, have been shown to directly degrade when introduced into the body. Their protection from biodegradation, before their release at the target site, can be ensured by their encapsulation inside hydrophobic shells made of polymers, such as zein protein [11]. Hydrophilic and hydrophobic drugs have also been protected within composite capsule shells and their organic cores, respectively [36]. Similarly, many such strategies have been employed for the protection of the cargo inside the polymeric capsules.

*Increasing cargo bioavailability.* Encapsulation in polymeric capsules has also been applied to increase the bioavailability and dissolution rate of the cargo. Such cargo materials are usually hydrophobic, and their better absorption in the body requires structural modifications and changes in their degree of crystallinity. These modifications can be introduced by making biphasic, amorphous solid dispersions (ASDs) of the hydrophobic, crystalline cargo with a natural polymeric material [37], or by changing the microenvironment of the encapsulated cargo. Such a strategy involves the entrapment of the cargo in a polymer matrix or an acidic compound, such as citric acid. ASDs have been made to serve as solid cores encapsulated within protein microcapsules [12,21]. ASDs of various drugs have also been made in composition with a variety of natural polymers, such as gaur gum, xanthan gum, and acacia [38]. The encapsulation of ASDs in hydrophilic capsules also ensures the enhanced bioavailability of the cargo.

*Carriers as cell-culture platforms.* Hollow core capsules and porous spheres have served as 3D culture platforms/scaffolds for various types of cells for their better integration into the body at the tissue lesion-affected area and tissue regeneration. Depending upon the site of the lesion and type of tissue, various natural polymers can be selected for the synthesis of capsules and spheres that may serve as platforms for cell and tissue culture. Porous microspheres have been shown to provide a larger surface area to serve as effective cell culture platforms. It has been shown that spheres with pore diameter ≥20 μm are suitable for cell culture inside the sphere pores [26]. Microcapsules and porous spheres made of various natural polymers, such as collagen [13], gelatin [23], silk fibroin [39], pectin [40], chitosan/gellan gum [41], chondroitin sulfate, alginate, etc., have been shown to serve as excellent scaffold materials for cell culture, especially in bone tissue regeneration strategies. In addition, these capsules and spheres have been supplied with bioactive strategies that assist in cell attachment, proliferation, and differentiation. Thus, cell carriers can not only be made to act as 3D cell culture platforms but also induce cell differentiation to assist in easy, fast, and better integration of cultured cells at lesion sites.

*Blood-vessel embolization.* Microcapsules of natural polymers have also been made to enable blood vessel embolization, a strategy concerned with the deliberate blockage of blood flow in the vessels and arteries to cut off nutrition and oxygen supply of tumor [14,42]. Biocompatible, biodegradation, and non-toxic properties of natural polymers are advantageous for this strategy. An ideal embolizing agent must possess good mechanical strength and be of appropriate size that can adapt to the target blood vessel diameter. Moreover, it should be visible under X-rays and potentially impair angiogenesis [14]. Controllable degradation, good biocompatibility, and blood compatibility are other essential properties of an embolizing agent. Microcapsules and spheres of chitosan [43], gelatin [44], starch [45], alginate [46], etc. [47], have been used as embolizing agents in the treatment of various cancer therapies. In addition to their embolizing effect, these agents can also act as carriers for anti-cancer drugs (to act on cancer cells synergistically). 

Various micro- and nano-capsule core–shell configurations have enabled the introduction of multi-functionalities in the capsules, thus employing one or more of the aforementioned strategies. For instance, microcapsules have been developed to enable simultaneous functions of sustained-release of drug and blood vessel embolization [14,43], targeted delivery and sustained-release [10,35], sustained-release, and tissue regeneration by cell delivery [26] and the like.

#### 2.1.2. Modes of Encapsulation

A cargo is encapsulated into the micro- or nano-capsules either during (in-process encapsulation) or after the capsule synthesis (post-synthesis encapsulation) [39], depending upon the type of application or design convenience. In-process encapsulation involves the introduction of cargo in the appropriate precursor solutions before applying one of the capsule synthesis techniques described in the following sub-section. Post-synthesis encapsulation is mainly achieved by incubating the capsules in the cargo solutions, leading to their absorption by the capsules. The cargo can be introduced at the desired location in the capsules using both ways. 

The APC can be dissolved or dispersed in either the core or the shell matrix (Figure 2). In the case of liquid-core capsules, the APC can either be dissolved in an oily carrier [12] or exist as an aqueous core [25]. Alternatively, it can also be encapsulated by the polymeric shell in its free form. In both cases, the APC exists as a reservoir inside the capsule core. In a solid core capsule, the APC can be entrapped in the solid core as a matrix system [11].

### 2.2. Synthesis Approaches and Mechanisms of Carrier Formation

Over the decades, many techniques for synthesizing natural polymeric micro- and nano-capsules and spheres, involving various chemical, physical, or physiochemical processes, have been developed and reviewed in detail by many authors [48,49,50,51,52,53]. This section, therefore, refrains from discussing general procedural technicalities in detail. Instead, it takes a closer look at the structural configuration-specific synthesis approaches, processes involved, and modifications introduced in the preparation of natural polymer-based spherical capsules with liquid/hollow/solid cores and porous microspheres. Mechanisms and interactions involved in capsule formation have also been discussed wherever necessary and possible. Generally, polymeric micro- and nano-capsule synthesis techniques follow the approaches and processes discussed herewith. 

#### 2.2.1. Solid Templating

**Figure 3:**
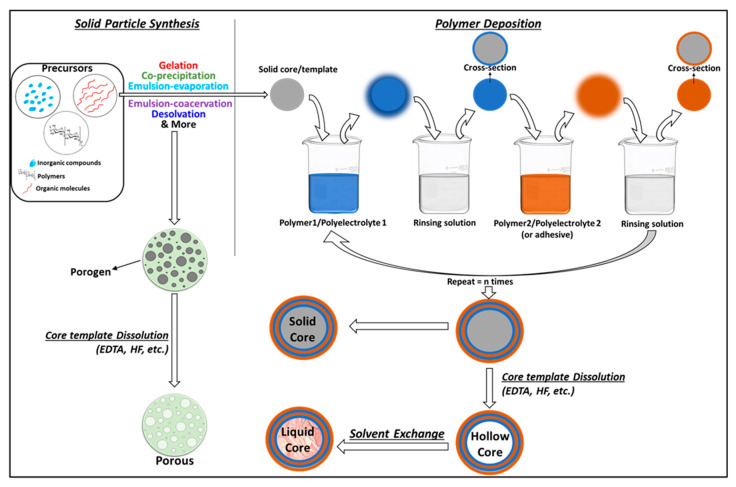
Schematic diagram of the solid templating approach.

This route involves the deposition of layer/s of polymer over solid micro- or nanoparticles of oxides, carbonates (CaCO_3_, MnCO_3_, or CdCO_3_), metallic particles, or natural [8] or synthetic polymers to yield core–shell capsules. Polyelectrolytes with opposite charges can be easily alternatively deposited to form multiwalled capsules. The deposition is usually carried out by dipping the core template alternately in different polymeric solutions to achieve the desired number of polymeric shell layers and is facilitated by non-covalent and covalent interactions between the core and first polymer layer, as well as the consecutive polymer–polymer layers. The method, thus, enables the formation of layers of different polymers, capable of carrying a variety of drugs possessing different physical and chemical properties. The drugs can be introduced into the polymeric layers during layer assembly and into the core via co-precipitation during the core formation (or after the synthesis of the system, through absorption). Figure 3 presents a schema of the general procedure involved in the solid templating technique. Solid core multiwalled, as well as hollow core multiwalled micro- and nano-capsules, can be made using this process (refer to Table 2 for examples). Solid templating is a promising approach that provides more refined control over the capsule size, thickness, functionalities, encapsulation mode, type of solid core, and morphologies. 

Preparation of hollow core capsules is straightforward by solid templating. Typically, natural polymers are deposited over a sacrificial template core to create single, double, or multilayer shells solid core microcapsules. After the deposition of the shell layer/s, the template is dissolved to give a hollow core. Many types of materials have been used as sacrificial template cores, amongst which silica and calcium carbonate [51] nano- or microparticles are the most common. The core template dissolution is carried out by immersing the capsules in a chelating solvent, such as 8% hydrofluoric acid [54] and ethylenediaminetetraacetic acid (EDTA) [55]. During this process, the solvent molecules diffuse into the capsules to dissolve the solid core. It is significant to note that the core template must be completely dissolved. To ensure that, the core removal step is repeated multiple times. However, it has been shown that the core chelating solvents are not thoroughly removed during the capsule purification step, which may pose toxicity-related issues for biological applications. Yitayew et al. gave a proof-of-concept, using endotoxin-free cell lines as sacrificial template cores to mitigate these issues. They used live *E. coli* DH5 cells as a sacrificial template for synthesizing hollow core chitosan–alginate multiwalled capsules [56]. The microcapsules were dispersed in lysis buffer (0.1% Triton X-100, 2 mM EDTA in 10 mM Tris-pH8) overnight and washed with acetic acid buffer to remove the template cells. It is also worth noting that the core-removal step can cause shell deformities and engage in undesired reactions with the APC [57]. As mentioned earlier, the core materials in solid core micro- and nano-capsules may carry functionalities such as drug entrapment by the co-precipitation of the APC into a solid polymeric core [14], magnetically guided systems involving paramagnetic nano- or microparticles as solid cores [58], as implantable capsules with titanium microparticle core [59], and several more. Oily core polymeric shell capsules have also been indirectly prepared by using solid templating to prepare hollow core capsules and filling the capsule core with an organic solvent by solvent exchange [60]. Solid templating can also be employed to prepare porous spheres. To do so, solid template particles (also known as porogen) are dispersed in the aqueous or oil phase containing the dissolved polymer [61]. The obtained phase with dispersed solid porogen particles is then emulsified with the water–oil phase to obtain porogen-containing microspheres [62]. The template moieties are then dissolved to give porous spheres [63,64]. Different types of solid porogens can be employed, including polymer particles, such as polystyrene [62] and gelatin [64]. Various examples of porous spheres prepared by solid templating are presented in Table 3.

The surface charge of the cores (solid cores or sacrificial templates) is modified to facilitate attractive forces and interactions for the deposition of polymeric layers [65]. Shell-forming polymeric materials are more often oppositely charged polyelectrolytes. The most commonly used oppositely-charged (positive-negative) natural polyelectrolyte pairs for LbL deposition are chitosan–alginate [65], chitosan–hyaluronic acid [55,59], gelatin–epigallocatechin gallate [36], and BSA polycation–alginate [17]. Traditionally driven by electrostatic attractions between the opposite charges, these oppositely-charged polyelectrolytes sequentially self-assemble around the core during the dipping process to form micro- or nano-capsules [17,66]. The self-assembly can also be facilitated by hydrogen bonding between neutral polymers, as well as charged polyelectrolytes [67,68], generally by introducing modifications. Manna et al. used adenine modified neutral chitosan (CS) and thymine modified negative hyaluronic acid (HA) polyelectrolyte to mimic DNA base-pairing between adenine and thymine, enabling the self-assembly of these polymers into thin layers [68]. In another study, silk fibroin multilayers were deposited on a silica template using tannic acid (TA) as an adhesive between the silk layers aided by hydrogen bonding between the protonated hydroxyl group of TA and carbonyl groups present in silk fibroin [54]. These hydrogen-bonded capsule layers are often exploited to enable pH-stimulated cargo release from the capsules. 

However, non-covalent interactions, such as hydrogen bonding and electrostatic attractions, are not robust enough to sustain the drastic pH differences and variations in ionic strengths across different biological environments, which may result in premature disassembly of capsules, unintentional release of cargo, aggregation, and fusion of multilayers, resulting in the loss of their multi-functionalities [69]. To remedy this, covalent interactions have been induced between polyelectrolyte layers, before or after their assembly over a solid core. Post-assembly covalent interactions between the polymeric layers are usually established by incubating the capsules in a solution containing cross-linkers, such as genipin and glutaraldehyde [65,70]. Glutaraldehyde crosslinks hydroxyl groups and amino groups in natural polymeric layers of the capsules [71]. To avoid the use of external crosslinkers, modified polyelectrolytes have been used to facilitate crosslinking. Oxidized sodium alginate (OSA) and CS were covalently assembled by crosslinking between the aldehyde groups of OSA and the amino groups of CS [72]. In another study, chitosan and hyaluronic acid were thiolated before their assembly into alternative layers on the CaCO_3_ template. Disulfide cross-linking between thiolated polyelectrolyte layers was induced post-assembly, mediated by horseradish peroxidase and tyramine hydrochloride [55]. Liu reviewed and classified several methods employed to stabilize LbL assembled core–shell capsules of various synthetic and natural polymers [69]. Apart from covalent cross-linking, they described surface concealing as one of the methods to protect the capsules from adhesion and collapse, while retaining their ionic-responsive properties. It is noteworthy that, although covalently assembled and stabilized hollow and solid core shell capsules can sustain drastic pH and ionic strength changes, non-covalent interactions can facilitate the stimuli-responsive release of the cargo at the target with characteristic pH. Hence, a tradeoff must be achieved between the two. We believe that surface concealing of the capsule shell layer/s may prove worthy on such occasions.

#### 2.2.2. Emulsion Templating

**Figure 4:**
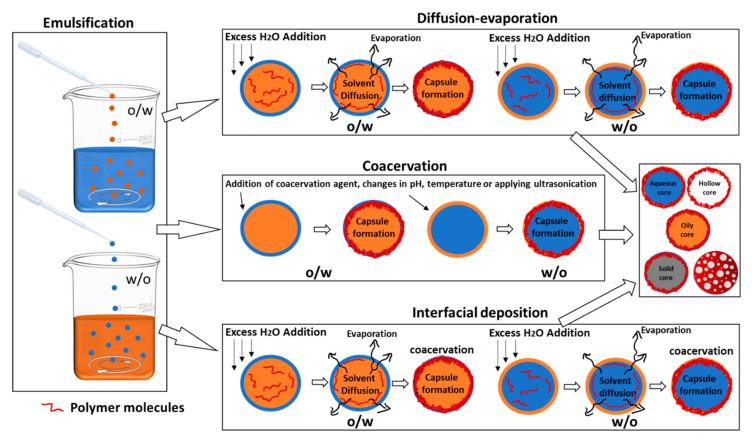
Schematic diagram of emulsion templating approach.

These methods utilize micro- or nano-emulsions between two or more types of mutually immiscible solvents as templates for polymeric capsule growth. Depending upon their solubility, the type of capsule configuration required and mode of APC encapsulation, the natural polymer, the APC, stabilizers, cross-linkers, and/or surfactants are dissolved in the appropriate solvents. Two types of emulsions can be achieved, i.e., (a) single emulsion: water in oil (w/o), oil in water (o/w); and (b) double emulsion: w/o emulsion, dispersed in water to give w/o/w or vice versa to give o/w/o. The resulting emulsion is then subjected to different types of chemical, physical, or physiochemical processes, such as diffusion-evaporation, coacervation, ultrasonication, crosslinking, interfacial deposition, solidification, spray-drying, freeze-drying, etc., to achieve stable biopolymeric capsules having liquid/solid/hollow cores. The steps involving the emulsion formation and diffusion-evaporation/coacervation/interfacial deposition are modified as needed. A procedural schema of the emulsion templating technique is represented in Figure 4. It is important to remember that emulsion templating can be used to achieve both spheres and capsule configurations by introducing variations during the synthesis procedure. However, we will mainly focus on the synthesis of core–shell capsule configurations and porous spheres. Various recent examples of capsules prepared using the emulsion templating technique are listed in Table 4. Porous spheres synthesis, using emulsion templating, involves the addition of porogen such as effervescent salts like ammonium bicarbonate or other inorganic salts (like sodium chloride) [73], in the appropriate phase, prior to the emulsion formation [64]. Ice crystals have also been employed as porogens. During the procedure, the polymer-containing emulsion is rapidly cooled to freezing temperatures to form ice crystals before initiating crosslinking or polymer precipitation. Ice crystals are then removed by sublimation or vacuum drying to produce highly porous polymeric spheres [26,74,75]. Table 3 lists recent examples of porous spheres prepared using the emulsion templating technique.

##### Emulsion–Diffusion–Evaporation

This method involves a mixture of partially water-miscible solvent (such as ethanol, acetone, or ethyl acetate), water, and an immiscible solvent (oils such as soyabean oil, Miglyol, or oleic oil). The process usually requires the preparation of mutually saturated organic solvent and aqueous solution [76]. For an oily core capsule formation, the organic phase includes preparing the APC for encapsulation, an optional hydrophobic stabilizer, and a water-immiscible oil/organic phase, dissolved in the water-saturated partially water-miscible organic solvent. The external aqueous phase consists of the polymeric shell material and one or several hydrophilic stabilizers, dissolved in the solvent-saturated water. To prepare a hollow core capsule, similar steps to those mentioned above are used, except for adding a water-immiscible oil/organic solvent in the organic phase. An o/w emulsion is made by introducing the water-saturated organic phase into the solvent-saturated aqueous phase, under constant stirring. The emulsion is then subjected to diffusion and/or evaporation as follows: 

(a) Fast diffusion, by dilution with water: an excess of water is added to the emulsion, such that the partially-water miscible organic solvent from the organic droplets of the emulsion diffuses out, leaving behind the polymer-stabilized capsules. The amount of water required should be enough to diffuse out and dissolve the inner, partially water-miscible organic phase. 

(b) Further, the solvent-dissolved diluted water is removed by evaporation under reduced pressure. Sometimes, the undiluted emulsion is directly subjected to the rapid displacement of the organic solvent from the internal to the external phase by evaporation under reduced pressure. 

Similarly, aqueous core capsules can be prepared by making w/o emulsions. The unwanted solvent, the excess of untrapped APC, and the stabilizers are then eliminated by reduced pressure, dialysis, ultracentrifugation, or crossflow filtration. Preparation of porous spheres through this approach involves the formation of double emulsions and depends upon the rate of solvent diffusion from the inner phase to the outer phase and its evaporation [64]. While diffusing from the inner to the outer phase, the volatile solvent evaporates, leaving a porous polymer matrix behind.

##### Emulsion-Coacervation

The process involves three main steps: (a) preparation of an emulsion (o/w or w/o) (b) coacervation, which involves the separation of the liquid phases in a colloidal solution brought about either chemically (by changing the pH, temperature, or ionic environment) or physically (by ultrasonication, to encapsulate the dispersed core material), and (c) stabilization of the polymer as shells by physically- or chemically-induced crosslinking. Both single and double emulsions can be coacervated using this technique. Usually, a double emulsion is used for the entrapment of hydrophilic drugs in the capsule’s core to ensure their efficient encapsulation. Additionally, it is easier to prepare aqueous core capsules using double emulsions. During the chemical coacervation step, the core and shell materials (polymers and APCs), dissolved in different/same solvents, are precipitated around the emulsion droplets by changing the pH of the system (with the addition of an acid or a base), lowering/increasing the temperature, or salting out, a process wherein the addition of appropriate ionic salts brings about a decrease in the solubility of the non-electrolytic biomolecules in the system. Simple coacervation is usually carried out for a system with one type of non-electrolytic biopolymeric precursor solution, which is usually coacervated using the salting out process [77]. Complex coacervation involves oppositely-charged polyelectrolytes of two or more biopolymers, and changing the pH usually brings about coacervation between them [50]. Stabilization of the polymeric shell, after its precipitation around the core material, during the chemical coacervation, is carried out by adding external crosslinkers, such as divinyl sulfone, 2,3-dibromopropanol, glutaraldehyde, etc. The concentration of the cross-linkers determines the thickness of the polymeric shell of the capsules and can be tweaked to achieve the desired thickness [52]. Physical coacervation can be induced by initiating electrostatic interactions between polyelectrolytes of different polymeric materials or polymeric sidechains of the same type of polymer or oxidative cross-linking. Electrostatic attractive forces between various polyelectrolytes of opposite surface charge have been utilized to enable physical coacervation to form capsules of two or more types of polymers [52].

**Figure 5:**
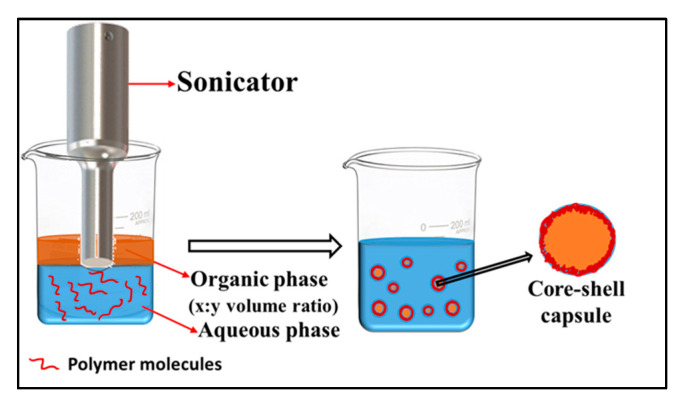
Schematic diagram of ultrasonication-assisted emulsification-coacervation.

*Ultrasonication-assisted emulsification-coacervation.* Physically-induced coacervation generally involves utilizing physical forces, such as ultrasonication, without the need for external cross-linkers. In a typical process, ultrasonication is applied at the oil–water interface when an aqueous solution of a polymer is overlayered with an organic phase, such as oil or other water-immiscible organic solvents (see Figure 5). Depending upon its solubility and desired location, the APC can be dissolved either in the aqueous or the organic phase. After a few minutes of sonication (~3 min), an o/w emulsion is formed and coacervated, due to various physical phenomena, induced by sonication. The first liquid-filled protein microspheres, prepared by Suslick, were composed of bovine serum albumin (BSA) and were filled with air [78] or organic phases [79], such as n-dodecane, n-decane, n-hexane, cyclohexane, or toluene. Ultrasonic irradiation of human serum albumin (HSA) or hemoglobin (Hb) formed similar microspheres to those of BSA. Since then, ultrasonication has been increasingly utilized to synthesize capsules of both synthetic [80], as well as other natural polymers [81,82]. It has proven to be a facile, cost- and time-effective technique that enables highly efficient encapsulation of a variety of drugs/cargo in the shell, as well as the core of the capsules. Our group has utilized this technique to synthesize protein microcapsules of BSA [83], HSA, and egg albumin [84], encapsulating a variety of hydrophilic and hydrophobic cargo, including gemcitabine [20], ribonucleic acid (RNA) [16,35], rhodamine B [85], MSQ (12i) 1-methyl-4-(substituted) styryl-quinolinium derivative [85,86], etc.

During ultrasonication-assisted emulsification, the size of the protein microspheres depends on the nature of the oil–water interface, viscosity, surface tension, and hydrophobicity of the organic phase. The hydrophobicity of the material inside the protein microspheres determines the stability of the microspheres [87,88,89,90,91]. High viscosity leads to the formation of larger structures, which, in turn, results in a decrease in the stability and fraction of active material incorporated inside the microspheres. The ratio between the hydrophobic content and water phase also affects the stability and size of the capsules. A smaller ratio between the two leads to the formation of smaller capsules, which are less stable, as opposed to the ones formed at a larger ratio between the two. It has also been shown that an oil–water ratio of (>0.5) can cause phase inversion, giving a w/o emulsion [92]. The chemical and physical nature of the encapsulated material also affects the size of the microspheres as well as the hydrophobicity of the core of the capsule. In the case of proteins and polypeptides, the stability of the oil–water emulsions depends upon the protein sequence and the molecule size. The amphiphilic nature of proteins is also responsible for their self-assembly at the oil–water interface, thus stabilizing the emulsion [93]. Suslick found that protein microspheres are created only in the presence of oxygen or air [91]. He explained that the sonochemical process, which follows an implosive collapse of gas bubbles, produces OH· and H· radicals. These radicals form H_2_, H_2_O_2_, and, in the presence of O_2_, the superoxide radical HO_2_. Hydroxyl, superoxide, and peroxide radicals are all potential protein cross-linking agents. Suslick and co-workers proposed that cysteine, a sulfide-containing amino acid present in these three proteins, is oxidized by the superoxide radical. The microcapsules are held together by protein cross-linking through disulfide linkages. Silva et al. alternatively proposed that amphiphilic polymers, such as proteins, can form stable microcapsules, due to the presence of hydrophilic and hydrophobic moieties that align themselves at the oil–water emulsion interface, due to the high shear forces generated by ultrasonication, and are entirely independent of cysteine content in the protein [88]. This alignment can also induce changes in the secondary structure of proteins, such as that of silk fibroin, which experiences an increase in its β-sheet content. Additionally, the cavitation produced during ultrasonication induces thermal denaturation of proteins, which in turn assists in the formation of the microcapsules [87].

##### Emulsion-Interfacial Deposition

This technique involves a combination of diffusion-evaporation and coacervation after the formation of an emulsion. In a typical process, an organic solvent (with oil and/or partially water-miscible solvent), containing the dissolved APC and/or the dissolved polymer, is introduced drop-by-drop into an aqueous solution, under constant stirring. Subsequently, large volumes of water are added, such as in the emulsion–diffusion–evaporation method. This is done to draw out the partially miscible organic solvents from the emulsion droplets, thus driving the polymeric molecules inside the organic emulsion droplets to precipitate at the droplet interface under the suitable pH, temperature, or ionic conditions (similar to the chemical coacervation method) [51]. The particles are recovered and cleaned using centrifugation and filtration. Narrow size distribution is obtained. The technique does not require the usage of external high-energy sources. However, it is limited by drug solubility, given that hydrophilic drugs cannot be encapsulated using this technique. In addition, the removal of residual solvent is challenging. Other disadvantages include the requirement of extensive optimization of parameters, such as the salt type (and its concentration), intensive purification of the obtained particles, and possible incompatibility of the salts with the bioactive drugs. Aqueous core capsules have been prepared using this technique, wherein an aqueous phase containing acetone (lower boiling point than water) and the dissolved polymer is added to the oil phase to give w/o emulsion, followed by subsequent evaporation of acetone at reduced pressure and ambient temperature [94]. During the evaporation process, the dissolved polymer precipitates at the water–oil interface of the water droplet, due to the decrease in acetone concentration, and forms the polymeric capsules with an aqueous core. The rationale here is to utilize a polymer that is soluble in the water–acetone solution (but insoluble in pure water or oil). Hence, the choice of polymer is crucial. This may be why the aqueous-core capsules that are made of natural polymeric shell and prepared using this technique are hard to find.

##### Emulsion-Spray Drying

In a typical spray-drying method for capsule formation, the polymer/s and the APC are dissolved in appropriate solvents to form shell and core materials. The core material is introduced into the shell material, and the resulting emulsion is dispersed as ultrafine droplets through a nozzle in a hot air flow [12]. The solvent evaporates instantaneously, and the dried capsules are collected under low pressure in a dry airflow. Solid core capsules are easily synthesized using this technique. Porous spheres can also be synthesized by spraying polymer and porogen, followed by removing the porogen templates. Spray-drying is easy to perform, yields consistent capsule sizes, is scaled up effortlessly, and fully automated. However, the adhesion of material on the walls of the instrument, agglomeration, and nozzle clogging hinder the yield, leading to high maintenance costs. Additionally, it is hard to get capsules under the 100 microns size range [77]. 

#### 2.2.3. Other Techniques

##### Coextrusion–Coacervation

Precursor solutions of the core and shell material are fed into the concentric nozzles (of preset diameters) and extruded into a non-solvent (solidification liquid) at a specific rate to form core–shell droplets, which undergo coacervation to form core–shell capsules. Sometimes external crosslinkers are added to obtain stable capsules [95]. Porous microspheres have also been prepared using a similar approach of extrusion/injection of the polymeric solution into liquid nitrogen to form ice-crystals that act as porogens to give porous polymeric spherical matrices [39,96,97]. The size and shape of the capsules depend on the feeding rate, temperature, and type of precursor core–shell solvents, as well as the distance between the nozzle and the solidification liquid, its concentration, and surface tension [50]. Similar to spray drying, this technique is also limited by blockage of the nozzle and is high maintenance.

##### Microfluidics

The method involves the formation of emulsions (o/w or w/o) in various microfluidic devices. A microfluidic device is set with pre-requisite conditions, such as size, shape, and reproducibility. This allows the formation of carefully controlled polymeric capsules with entrapped drug molecules. However, it is not suitable for the synthesis of nano-sized capsules because of the inherent micron-length scale of the device. Different microfluidic systems, including T-mixer and co-flowing junction, hydrodynamic flow flowing, multi-inlet vortex mixers, staggered herringbone, and toroidal mixers, are used for achieving polymer particles or capsules of various sizes and shapes [97]. Using a microfluidics T-junction mixer, Mendes et al. produced hollow core polypeptide–polysaccharide (xanthan gum) microcapsules [98]. Porous microspheres have also been prepared with the assistance of microfluidics [99].

**Table 2:** Examples of capsules prepared by solid templating approach.

Polymer 1/Polyelectrolyte 1	Polymer 2/Polyelectrolyte 2	Solid Template/Core	Template Dissolving Agent	Template/Core Synthesis Method	Shell-Type and Deposition	APC and Location	EE (%)	Capsule Surface Charge (mV)	Template/Core Size and Capsule Size	Core-Polymer and Polymer–Polymer Interactions	Crosslinking between Core and Layers	Ref.
BSA polycation (+5.05 mV)	Alginate polyanion (−24.6 mV)	Template: amine modified-SiO_2_ (+11.8 mV)	NH_4_F/HF	Stöber process	Multiwalled (seven alternate layers of BSA and Alginate)	Betamethasone disodium phosphate (BSP); shell; post-synthesis introduction	56%	+5.05 mV	~128 nm; ~170 to188 nm	Non-covalent (hydrogen bonding, electrostatic, van der Waals, and hydrophobic interaction)	-	[17]
BSA	Tannic Acid	Template: CaCO_3_. Core: BSA	Ethyl-enediaminetetraacetic acid trisodium salt (EDTA)	Co-precipitation	Multiwalled (six bilayers of BSA/Tannic Acid)	Tetramethylrhodamine-isothiocyanate labeled BSA; core; co-precipitated with the solid template during synthesis	-	(−30 ± 1.9) mV	-	Hydrogen bonding	-	[33]
Silk fibroin (anionic)	Aminopropyl triethoxysilane (APTES) (cationic)	Template: polystyrene	N,N-dimethyl formamide (DMF)	-	Multiwalled (nine layers of Silk fibroin)	chlorin e6 (Ce6) and doxorubicin (DOX); shell; post-synthesis introduction	DOX = 80% Ce6 = 90%	−	~150 to 250 nm; ~230 nm	Electrostatic interactions	-	[8]
Silk fibroin	-	Solid core: poly(lactic-co-glycolic acid)	-	Single emulsion-solvent evaporation method	Single layer of silk fibroin	Simvastatin; Core; in-synthesis encapsulation	59.4% to 70.3%	-	~15.3 μm	Covalent bonding	Chemical crosslinking by Glutaraldehyde	[24]
calcium cross-linked k-carrageenan	k-carrageenan and chitosan polyelectrolyte complex	Template: CaCO_3._ Core: BSA	EDTA	Co-precipitation	Multiwalled	Curcumin; after core synthesis, before layer assembly	6.25 to 8%	-	-	Electrostatic interactions	-	[100]
Gelatin A	(−)-epigallocatechin gallate (EGCG)	Template: MnCO_3_	EDTA	-	Multiwalled (four layers)	-	-	−25 mV	~4.0 μm; ~4–5 μm	Non-covalent (hydrophobic and electrostatic interactions)	-	[36]
Chitosan polycation	Alginate Polyanion	Template: *E. coli* cells (−32.70 ± 3.2 mV)	Lysis buffer (0.1% Triton X-100, 2 mM EDTA in 10 mM Tris-pH8)	Cultured	Multiwalled (four bilayers of chitosan–alginate)	-	-	(−36.08 ± 8.8) mV	-	Electrostatic interactions	-	[56]
Thiolated-chitosan polycation	Thiolated-hyaluronic acid polyanion	Template: CaCO_3_ −15.8 mV	EDTA	Co-precipitation	Multiwalled (four bilayers of chitosan/hyaluronic acid)	BSA and Dextran; Core; Co-precipitated with the solid template during synthesis	20.2%	−11 to −25 mV	3.0 µm; 4 to 6 µm	Covalent interactions by disulfide bonding	Enzymatic crosslinking using horseradish peroxidase and tyramine hydrochloride	[55]
Chitosan	-	Solid; Ca-alginate	-	Extrusion	A single layer of chitosan	Insulin and probiotic cells; post-synthesis	-	-	--	-	Electrostatic interactions	[101]

**Table 3:** Examples of porous spheres prepared by solid & emulsion templating approach.

Polymer Matrix	Porogen	Preparation Method	Porogen Removal Process	Crosslinkers; Precipitants	APC	Pore Size	Sphere Size	Ref.
Silk fibroin	Ice crystals ~(−195 °C)	Microinjection into liquid nitrogen and freeze-drying	Sublimation	-	Basic fibroblast growth factor (bFGF)	1.5–7.0 µm	95 µm to 260 µm	[39]
	Ice crystals (−20 °C)	w/o emulsion, rapid cooling, and freeze-drying	Sublimation	-	Strontium	(20 ± 5) to (34.8 ± 6.5) μm	-	[26]
		Microinjection into liquid nitrogen and freeze-drying	Sublimation	Ethanol-assisted precipitation	-	0.3–10.7 μm	208.4–727.3 μm	[102]
Chitosan	Ice crystals (−20 °C)	w/o emulsion, low temperature, thermally-induced phase separation, and pH-assisted coacervation	Drying under vacuum	-	-	20–50 μm	ca. 150 μm	[74]
	Ice crystals ~(−195 °C)	Microinjection into liquid nitrogen and freeze-drying	Sublimation	Saturated sodium tripolyphosphate (STPP) crosslinker	-	<30 μm	<400 μm	[96]
Chitosan/poly(L-glutamic acid) (PLGA) polyelectrolyte complex	Ice crystals (−20 °C)	w/o emulsion, low temperature, thermally-induced phase separation	Drying	-	-	(47.5 ± 5.4) μm	250 μm	[75]
Collagen/cellulose	Solid polystyrene	w/o emulsion	Washing with acetone	n-butyl al-cohol as precipitant	BSA	~198.5 nm	8–12 μm	[62]
Alginate	NaCl	w/o emulsion, freeze drying	-	Calcium chloride as crosslinker	-	200–300 nm	~158 μm	[73]
Soy protein	CaCO_3_	Solid templating over porogen by incubation	Dissolution by EDTA	Transglutaminase as crosslinker	-	-	3–12 μm	[61]
Silk sericin and hydroxylapatite	Silk sericin	Nucleation and growth of hydroxyapatite, induced by the sericin template in simulated body fluid	-	-	Doxorubicin	-	1–3 μm	[103]

**Table 4:** Examples of capsules prepared by emulsion templating approach.

Polymer Shell	Core & Type	Template & Organic Solvent	Emulsion Type	Method	APC & Location	Interactions	Crosslinkers; Stabilizers; & Surfactants	Surface Charge	Size	Encapsulation Efficiency	Ref.
Human serum albumin (HSA)	Lauroglycol 90; oily	Lauroglycol 90; Acetone	o/w single emulsion	Diffusion-evaporation	Exemestane and hesperetin; core	Electrostatic interactions	None; 1:1w/w poloxamer/Tween 80 mixture; benzalkonium chloride	20.7 ± 1.26 mV	172.4 ± 8.6 nm	95–98%	[10]
Folic acid-functionalized HSA	Oily; dodecane	Dodecane	o/w single emulsion	Ultrasonic emulsification	-	Oxidative crosslinking	-	−20 mV	~440 nm	-	[22]
Wheat germ agglutinin-functionalized HSA	Biocompatible plant oils; oily	Almond oil, rapeseed oil, olive oil, and linseed oil	o/w single emulsion	Ultrasonic emulsification	-	Oxidative crosslinking	-	−12.4 ± 9.4 mV	(662.1 ± 7.6) nm to (862.2 ± 59.5 nm)	-	[104]
Fluorescently tagged bovine serum albumin (BSA) shell; Shell filled with PLGA and unsaturated fatty linoleic acid	Lecithin; aqueous	Dichloromethane and ethanol	w/o/w double emulsion	Double emulsion–evaporation	lipophilic paclitaxel in the oily shell and hydrophilic transcription factor p53 in the aqueous core	-	Pluronic F-68 & Lecithin	−36.4 mV	~180 nm	-	[25]
BSA	Soya bean oil; oily	Soya bean oil	o/w single emulsion	Ultrasonic emulsification	Ribonucleic acid (RNA); shell	Oxidative crosslinking	-	−40 meV	(0.5 μm to 2.5 μm)	~60%	[16]
Polyvinyl alcohol (PVA) functionalized-BSA								0 meV			
Polyethyleneimine (PEI) functionalized-BSA								+20 meV			
Silk fibroin	Sodium alginate; solid	Paraffin oil	w/o single emulsion	Emulsion-coacervation	-	Chemical crosslinking using glutaraldehyde	Span 80	-	Avg. 141.839 μm.	-	[14]
Collagen and PLGA layers	Hollow	Dichloromethane	o/w single emulsion	Emulsion–evaporation	MnO_2_ nanoparticles; shell	Carbodiimide initiated covalent crosslinking	Crosslinking facilitated by N-(3-Dimethylaminopropyl)-N′-ethyl carbodiimide hydrochloride (EDC), N-Hydroxysuccinimide (NHS); stabilizer: polyvinyl alcohol (PVA)	-	-	-	[18]
Anti-epidermal growth factor receptor (EGFR) modified-BSA	Dodecane; oily	dodecane	o/w single emulsion	Ultrasonic emulsification	Gemcitabine; shell	Oxidative crosslinking	-	-	~1.1 μm	30%	[20]
Whey protein isolate (WPI)	Sunflower oil; solid	Sunflower oil	o/w single emulsion	Spray- and freeze-drying	Vitamin E; core	-	-	-	~145.3 µm	89.3%	[12]
Gelatin	Citric acid; solid	Dichloromethane and ethanol	o/w single emulsion	Spray drying	Itraconazole; core	Physical crosslinking	-	-	-	-	[21]
Tetramethylrhodamine-isothiocyanate labeled-BSA, tannic acid, and BSA layers	Sunflower oil; oily	Sunflower oil	o/w single emulsion	Emulsion-coacervation	3,4,9,10-tetra-(hectoxy-carbonyl)-perylene (THCP); core	Hydrogen bonding between the shell layers	-	(−30 ± 1.9) mV	-	-	[33]
Chitosan	Soybean oil, oily	Soybean oil; benzyl benzoate	o/w single emulsion	Emulsion-microfluidic	Tea tree oil; core	Covalent interactions by chemical crosslinking	Terephthalaldehyde (TPA)	-	~106 μm	19.5–49.3%	[105]
Gelatin and gum arabica	Soybean oil; aqueous	Soybean oil	w/o/w double emulsion	Emulsion-complex coacervation	Sucralose; core	Covalent interactions	Lecithin		81 to 113 μm	43.04 to 89.44%	[106]
Folic acid-modified hyaluronic acid	Ethyl acetate; oily	Ethyl acetate	o/w single emulsion	Ultrasonication	Curcumin; core	Oxidative crosslinking	-	-	400 to 600 nm	91.3 to 93.2%	[107]
Soy protein and gum arabica	(80 vol% NEOBEE M5 + 20 vol% limonene); oily	80 vol% NEOBEE M5 + 20 vol% limo-nene	o/w single emulsion	Complex coacervation	-	Heat-induced gelation crosslinking	-	-	-	-	[108]
Pea protein isolate and sugar beat pectin	Hemp seed oil; oily	Hemp seed oil	o/w single emulsion	Complex coacervation, followed by spray-drying	Hempseed oil	pH-induced crosslinking	-	-	(12.80 ± 2.17) to (23.70 ± 1.23) μm	(79.65 ± 5.99) to (94.42 ± 6.63)%	[109]

## 3. Natural Polymer-Based Capsule Characterization

### 3.1. General Characteristics

#### 3.1.1. Size

One of the primary characteristics of any biomedical formulation is its operating size. It is a critical parameter that determines the suitability of the capsules to penetrate the target biological site, as well as its applicability arising from in-vivo pharmacokinetics [110]. In addition, the capsule size influences the drug-loading capacity, drug release rate and profile, and capsule stability [111]. Smaller capsules may provide a larger surface area for the entrapment of a surface-bound drug, leading to potentially higher loading capacity. However, a smaller core, achieved due to smaller size capsules, may not ensure sufficient loading capacity for a drug-loaded in the capsule core as a reservoir. Alternatively, a larger capsule, with a thicker shell (or multiwalled shell), can have a higher loading capacity for a shell-bound drug but may or may not have a higher loading capacity for a core-bound drug, in which case the core size is of paramount importance. A shell-bound drug releases at an accelerated rate from smaller size capsules, due to the increased surface area [111,112]. Larger polymeric capsules have been shown to degrade/dissolve faster than smaller capsules, due to bulk erosion [113]. However, it has also been previously shown that the particle size had a minimal effect on the polymer degradation rate [114]. Hence, it is safe to draw that the dependence of capsule degradation on its size may be system- and parameter-specific. 

Capsule size can be affected by the type of precursor polymer and its concentration [115], emulsion homogenization speed, agitation rate [116], type and concentration of the emulsifying agent [117], volume of the aqueous and the oil phase, size of the solid template/core, type and concentration of the surfactant, storage conditions, thickness of the polymeric shell, and synthesis technique employed. Valot et al. studied process the influence of process parameters on the size distribution of ethyl cellulose microcapsules synthesized, using the emulsion–evaporation technique [118]. They found that the mean capsule size decreases with the increase in the volume ratio of the dispersed organic phase to the continuous aqueous phase and an increase in the stirring rate. They also concluded that a decrease in the surfactant concentration leads to increased mean capsule size.

Size distribution measurements are usually performed using the dynamic light scattering (DLS) method, wherein the micro- or nano-capsules are dispersed in a solvent media during measurements. Size and morphological studies are also conducted using scanning electron microscopy (SEM) and transmission electron microscopy (TEM). However, care must be taken during sample preparation. We have observed that the liquid core microcapsules are prone to bursting during air drying and vacuum conditions in the SEM instrument. Lyophilization of the sample for ESEM measurements can be an option to avoid such a scenario.

#### 3.1.2. Stability 

The stability of micro- and nano-capsule concerns their storage, as well as operating in-vivo stability. After synthesis and purification, microspheres are either stored as colloidal solutions at lower temperatures, solid freeze-dried samples, lyophilized into powders, or in the form of spray-dried or vacuum-dried powders. Proper capsule storage ensures a better shelf-life of capsule formulations and their subsequent usage. Sonochemically prepared liquid-core human serum albumin capsules have shown to be stable for long-terms in suspension, as well as in freeze-dried conditions [104].

In-vivo stability of a capsule can be increased to avoid the initial burst release of the drug [31], which is usually an undesirable feature of a drug delivery formulation, and to extend the drug release rate. Moreover, the capsules can be stabilized and programmed to release drugs that target particular conditions, as in the case of stimuli-responsive release systems. 

#### 3.1.3. Moisture Content

Moisture content is an important physical property for the dried micro- and nano-capsules and spheres that influences the stability of the core after drying and affects the processibility, shelf-life, usability, and quality of the pharmaceutical product [119]. Furthermore, the maximum permissible moisture content in certain products depends on the guidelines established by regulatory bodies, such as the FDA. In general, products with moisture content between 3–10 g/100 g possess good storage stability [12]. 

Moisture content is determined using a thermogravimetric approach by measuring weight loss upon drying. Many moisture content measuring instruments are available. During a typical measurement procedure, the sample is heated, and the weight loss, due to moisture evaporation, is recorded [12].

#### 3.1.4. Surface Charge 

Another important property of any micro- or nano-capsule is its surface charge, which is usually determined by zeta potential measurements. The surface charge establishes the in-vivo capsule distribution and affects the drug release rate from the capsules. The surface charge can be modified using functionalizing polymers to enable targeted delivery of micro- and nano- capsules, for instance, to the cell nucleus [35]. 

#### 3.1.5. Encapsulation Efficiency

The efficiency of the drug encapsulation is calculated using the expression:

$$Encapsulation\;Efficiency\;\left(\%\right)=\frac{C_{t}-C_{un}\;}{C_{t}}\times 100\%$$

*C_t_* is the total concentration of the drug initially present in the precursor solution before capsule or sphere formation, and *C_un_* is the drug concentration measured in the residual precursor solution after the capsule or sphere formation.

#### 3.1.6. Drug-Loading Capacity

The drug-loading capacity is defined as the amount (weight) of drug-loaded per unit weight of micro- or nano-capsules and is calculated by the expression:

$$Loading\;capacity=\frac{W_{d}\;}{W_{c}}$$

in which *W_d_* is the total entrapped drug and *W_c_* is the total weight of the capsules. 

#### 3.1.7. Cytotoxicity

To determine the suitability and biocompatibility of capsule formulations, in-vitro cytotoxicity analysis is done in-vitro on tissue cells using cell viability and cytotoxicity assays [120]. These assays measure the cellular or metabolic changes associated with viable or nonviable cells and detect structural changes, such as loss of membrane integrity upon cell death or physiological and biochemical activities, indicative of living cells. Various types of cytotoxicity assays are available on the market, including MTT (methyl thiazolyl tetrazolium) and CCK-8 (Cell Counting Kit-8). The testing protocol for each is different and is explicitly defined by the assay manufacturers. In a typical procedure, the cells are incubated in 96-well plates at 37 °C, until adherent to the culture plates, followed by the addition of sterilized capsule suspensions. To these capsule-containing culture wells, prescribed volumes of cytotoxicity assay are added each day, incubated for 2 h, and scanned for absorbance at a particular wavelength to measure the optical density for counting the number of surviving cells and analyze their metabolic activity [14]. Zhou et al. describe various methods for cytotoxicity analysis of medical devices [120].

#### 3.1.8. Blood Compatibility

For any biomedical device or formulation, especially those intended to be introduced in-vivo through intravenous route and blood vessels, embolizing agents must have blood compatibility and should not cause hemolysis and blood coagulation. For blood compatibility analysis, the capsule formulation must undergo five stages of screening tests, which include thrombosis (blood clotting index, coagulation analysis, and platelets), hemolysis rate (nonhemolytic (0–2%), slightly hemolytic (2–5%), or hemolytic (>5%)) [121], and immunology testing [122].

#### 3.1.9. Flowability

Flow properties of the dried micro- or nano-capsule powder is an important parameter that establishes the powder quality. Usually, flow properties are analyzed by calculating the bulk and the tapped densities of a powdered sample. The procedure involves transferring a measured amount (*m*) of the powdered sample into a calibrated measuring cylinder and noting the bulk volume (*V_L_*) occupied by the powder to calculate the bulk density, $\rho_{b}$ by $\frac{m}{V_{L}}$. After this, the cylinder with the *m* amount of powdered sample is manually tapped for a certain amount of time to reach the tapped volume *V_T_* for calculating the tapped density, $\rho_{T}$ by $\frac{m}{V_{T}}$. The flowability of the power is then indirectly predicted using

$$\mathrm{Carr}’s\;\mathrm{index}\;\left(\%\right)=\frac{\rho_{T}-\rho_{b}}{\rho_{T}}\times 100$$

$$\mathrm{Hausner}\;\mathrm{Ratio}\;=\frac{\rho_{T}}{\rho_{b}}$$

Carr’s index ratings up to 10% are deemed excellent, between 10–15% are good, 16–20% are poor, 32–37% are very poor, and greater than 38% are abysmal. A Hausner ratio ≤1.25 indicates that the powdered sample is free-flowing, while a ratio ≥1.25 indicates poor flowability [12].

#### 3.1.10. Pore Size and Porosity

Depending upon the pore diameter size, micro- and nano-spheres can be microporous (<2 nm), mesoporous (2–50 nm), or macroporous (>200 nm). The pore size can be measured during morphological analysis using SEM, TEM, or confocal laser scanning microscopy. Porosity is the ratio between the pore volume and total volume of the microsphere. It can be calculated using a variety of methods [64].

### 3.2. Function-Specific Characteristics

#### 3.2.1. Drug Release and Kinetics

To understand the release behavior of the drug from a sustained-release capsule formulation, it is essential to study its release kinetics in-vitro. This is usually done by dispersing the drug-loaded capsule formulations in a release media under constant stirring and by measuring the drug concentrations in the release media at set time intervals. Conditions, such as the selection of proper release media, pH, temperature, and stirring speed, must be maintained and monitored throughout the in-vitro release experiments. The in-vitro release media is generally composed of the route- and target-specific biomimicking fluids at various pH values and bodily temperature (~37 °C). For example, orally administered capsule formulations are tested in-vitro in the gastrointestinal-mimicking release media. However, simulating exact in-vivo conditions is difficult. 

D’Souza reviewed various in-vitro drug release study methods [123], including ‘sample and separate’, ‘continuous flow’, and ‘dialysis method’. The ‘sample and separate’ method involves retrieving a certain amount of sample from the release media at certain time-intervals, separating the retrieved sample from capsules (via filtration, ultrafiltration, centrifugation, ultra-centrifugation, or their combination), and, finally, measuring the drug concentration in the filtrate or/and the evaluating the filtered capsules. This method, although straightforward, poses many challenges, including the clogging of filters during filtration and absorption of the drug molecules into the filters. We also faced similar challenges during drug release studies from organic-core BSA microcapsules [85]. In addition, we observed that BSA microcapsules ruptured several times during sample ultrafiltration, which resulted in the premature drug release in the filtrate leading. In a continuous flow method, the release media flows through a column containing immobilized drug-loaded capsule formulation, and the effluent is collected and monitored by detectors. Several types of apparatus are available for the continuous flow method. However, it is a costly method and requires complicated set-up assembly. The dialysis method is straightforward. Generally, the sample is placed in a dialyzing membrane and suspended into the release media. Samples are retrieved from the release media and analyzed. The method is simple and advantageous over the ‘sample and separate’ and ‘continuous flow’ methods, with the exception that a few drugs can bind to the dialysis membrane, affecting their concentration in the release media. In addition, the behavior of the dialysis membrane in the release media must be monitored prior to their employment for drug release studies. Finally, the drug release concentration in the release media vs. time profile is generated and compared to theoretical and computational models to predict the drug release behavior from the capsules and ascertain the underlaying release mechanisms.

The drug release process typically involves the migration of drug molecules from their initial location in the capsule to the external surface of the capsule and then, eventually, into an in-vitro release media or at the in-vivo target site. The movement and release of the drug via this route are facilitated by various mechanisms, which are briefly discussed below [30,124]. In-vivo drug release is usually governed by a combination of two or more of these mechanisms, depending upon the type and design of the capsules or the spheres. 

*Diffusion.* This process involves the mass transfer of the molecules of a substance (solute) from one part of a system or solution to another, driven by the solute concentration gradient [125]. In other words, it is the movement of solute molecules from their higher concentration to their lower concentration in a solution, as long as this concentration gradient is maintained. After the concentration difference is equalized, the system reaches a state of equilibrium where no more solute diffusion from one part of the system to another takes place. This mass transfer of molecules is facilitated by thermal and Brownian motion, which results in random and repeated collisions between molecules. Usually, in a gradient of solute concentration, not all the solute molecules have a preference to move in one direction. Hence, while studying mass transfer by diffusion, a solution is divided into volume groups of solute molecules [30]. One group of molecules may move in one direction, while another group in the reverse direction. If the concentration of the first volume group is more than the second one, overall, more particles will move from the first group to the second, leading to a net flow of molecules from their higher concentration in group one to their lower concentration in group two. For releasing from a polymeric capsule, the drug molecules must diffuse from their initial position (inside the core drug reservoir or matrix, or the polymer matrix) to the outer surface of the polymer matrix and, eventually, into the release media.

*Erosion.* Drug release, by polymeric capsules, sometimes involves the erosion or disintegration of the polymer matrix by the kinetic degradation of the appropriate links between polymer–polymer molecules or polymer–APC molecules, due to the hydrolysis of bonds [126]. The hydrolysis of a bond depends upon the local environment (acidic or basic). In a drug reservoir system, erosion-controlled drug release occurs when the polymer matrix degrades, releasing the APC that it physically encapsulates. In the case of a matrix system, the APC is usually chemically linked to either the polymeric shell or the core and is released after the breakage of those chemical links, accompanying the degradation of the matrix. Erosion can occur at the surface [127], or in bulk [128], of the capsules. When water invasion is slow and the hydrolysis of polymeric bonds is rapid, surface erosion occurs, which reduces system dimensions [30]. In a matrix-type system, the surface erosion of the polymeric SDDS is accompanied by the release of the APC molecules. When water invades the SDDS more rapidly throughout the system than the hydrolysis of the surface bonds, several polymeric chains are broken, leading to the bulk erosion of the system. During the bulk erosion, the drug is initially released from the system through the surface and pores. This initial release is followed by a dormant stage (almost no drug release), where broken polymer chains, triggered by water invasion, form crystallites that are resilient against hydrolysis. Finally, the drug is released rapidly, due to the accelerated degradation of the polymer and polymeric crystallites, due to autocatalysis.

*Osmosis.* Osmosis involves the movement of solvent (biological fluid) from its higher concentration (i.e., lower concentration of the solute) to its lower concentration (i.e., the higher concentration of the solute) through a semi-permeable membrane, which allows the transport of smaller solvent molecules into the system but prevents bigger solute molecules from leaving the system. The process is controlled by osmotic pressure, which develops when two solutions of different solute concentrations are separated by a semi-permeable membrane [30]. The higher the osmotic pressure, the higher the chemical potential, which leads to an increased rate of transport of the solvent molecules through the semi-permeable membrane. In osmotically-driven drug release, the polymer matrix of the capsules or spheres acts as a semi-permeable membrane. Due to the built-up osmotic pressure, the water outside the DDC/S starts to permeate the capsule polymer matrix, resulting in its hydration and swelling. Eventually, due to the permeation of water molecules into the matrix, the drug (solute) concentration inside the SDDC starts lowering, which results in a decrease in the osmotic pressure. The hydration and swelling of the polymer matrix result in the matrix becoming partially permeable to the drug molecules, which decreases the osmotic pressure and drives the drug molecules to slowly escape the system through the now partially permeable polymer matrix of the SDDC [129]. The process is repeated alternatively on both sides of the polymer matrix on account of osmotic pressure and chemical potential, leading to a slow and controlled release of the drug. The rate of osmotic flow depends upon the concentration and nature of the drug, temperature, and hydraulic permeability of the polymer matrix. 

*Swelling.* Swelling of the polymeric membrane of an SDDC usually depends upon the hydrophilic behavior of the polymer or water–molecule interaction [130]. When the polymer is surrounded by water, the polymeric network expands because water enters the DDC rapidly, as opposed to polymer dissolution, which is slow. This leads to the swelling of the polymeric shell. The mechanism is similar to swelling, in the case of osmotically driven drug release from an SDDC. The primary parameters that control swelling are ionic content, cross-link content, and hydrophilic content of the polymeric shell.

*Partitioning.* DDCs are usually made of one or more polymers of different affinities and polarities from the APC they contain [30]. Hydrophilic drugs partition themselves in the aqueous phase hydrophilic moieties of the DDC, whereas the hydrophobic drugs tend to reside in the organic phase or hydrophobic moieties of the DDC. In order to be released from the DDC, the drug molecules travel through mediums of different affinities (hydrophilic or hydrophobic polymers) at different rates, depending upon their relative concentration and affinity to each phase. This affinity is defined as a partition coefficient, which is the ratio of drug solubilities in the two phases and describes the relative frequency with which the drug moves from one medium to another.

#### 3.2.2. Swelling Ratio

The diameter of the micro- and nano-capsules is measured before and after the swelling of the capsules. During swelling experiments, the capsules are dispersed in an aqueous media under stirring at varying pH and temperatures conditions [14]. Their diameters are measured at each interval of time, and the swelling ratio (%) is calculated using the equation:

(1) $$\frac{D_{t}-D_{0}}{D_{0}}\times 100$$

where, $D_{0}$ is the initial diameter and $D_{t}$ is the diameter of the capsules after swelling at time (t). It is vital to build a swelling ratio profile prior to in-vivo testing, in order to understand the swelling behavior of micro- and nano-capsules, especially for their utility as embolizing agents operating at different diameters of blood vessels, as well as osmosis-controlled drug release systems. 

#### 3.2.3. Cell Survival Number

For determining the efficacy of the capsule as a protective enclosure to probiotic bacterial cells against the harsh gastric environment, in-vitro incubation of cell-encapsulating capsules and free cells in a simulated gastric fluid (SGF) is carried out for a set period to evaluate the cell survival number [11]. 

#### 3.2.4. In-Vivo Bioavailability

Capsules prepared for aiding the solubility characteristics of the encapsulated drug are tested, in comparison to the unencapsulated free drug, for its in-vivo oral bioavailability. The procedure involves live subjects (such as male or female rats in a similar weight range), divided into test and control groups. A certain amount (by weight of the live subjects) of drug-encapsulating capsules and the free drug are administered orally in the test and the control groups, respectively. Fixed volumes of blood samples are then drawn from the test and control groups at fixed time intervals (*t*_0_, *t*_1_, *…*, *t_n_*), through the experimentally preferred vein type (for example, the retro-orbital, the saphenous vein, or the tail vein in rats) [131]. Blood samples from a second control group of live subjects, to which no drug is administered, can also be studied for conducting an accurate evaluation. The collected blood samples from each group are analyzed for the blood plasma drug concentrations. Pharmacological analyses are carried out by generating the mean plasma concentrations of drug vs. time profile and analyzing the maximum plasma concentration (*C_max_*) at the time (*t_max_*) and area under the curve (AUC), to evaluate drug bioavailability from free drug and capsule-encapsulated drug [12]. 

#### 3.2.5. Dissolution Profile of the Capsules

The dissolution profile of a capsule formulation is built based on in-vitro experiments, which usually involve incubating the capsules in water/simulated gastric fluids over a definite period [12]. In such as case, the dissolution behavior is evaluated by observing the change in the absorbance intensity and optical density with time at the absorbance frequency of the capsule-forming polymer. The dissolution profile of capsule formulations reflects the capsule erosion over time in the release media and, as a result, indicates its biodegradation and elimination from the body, and affects the release behavior of the encapsulated APC. 

## 4. Recent Advances in Protein-Based Spherical Capsules towards Biomedical Applications

In the past few decades, various types of animal- and plant-based proteins and peptides have been studied for their use as drug and growth factor carriers, embolizing agents, and cell culture platforms, in order to enable sustained drug release, protection from the biological environment, enhanced bioavailability, targeted delivery, pH- and temperature-responsive release, embolization of blood vessels, better cell integration into the body and toxicity moderation. Table 1 lists various natural polymers (biopolymers), including proteins, that have been utilized to develop spherical capsules in biomedical applications. The interest in protein-based drug carriers stems from several of their advantages, namely higher biocompatibility and lower toxicity, biodegradability, high drug-binding capacity leading to a good drug-loading efficiency, possibility of straightforward and cheaper production due to their abundance in nature, the feasibility of structural modifications, due to the presence of several functional groups, non-immunogenicity, etc. Protein-based systems in the form of hydrogels, micro and nanoparticles, micro- and nanocapsules, implants, microneedles, bio-adhesives, fibers, rods, and films have been developed and tested for various applications in cancer therapy, nutritional therapy, diabetes, bone diseases, neurological conditions, and stem cell therapy. Spheres and capsules made of animal- and plant-proteins having liquid (organic or aqueous), hollow and solid cores have been developed for the above applications (see Table 5 and Table 6). 

In addition, the past decade has seen a considerable evolution of composite capsules made of two or more proteins, protein–polymer composite capsules, and composite capsules of protein conjugated with other materials, such as ceramics and metallic nanoparticles. Protein capsules have also been functionalized using other polymers to develop drug delivery formulations for targeted delivery. Herein, we focus on the advances made in the past decade towards developing micrometric and nanometric capsules with liquid, solid, and hollow core encapsulated by shells made of functionalized proteins and protein–protein composites, protein–polymer composites, protein composites with other materials, and multiwalled capsules. Our discussion revolves around protein capsules and spheres developed for biomedical applications, under the designing aspects discussed hitherto.

### 4.1. Liquid-Core Protein-Shell Capsules

With the purpose of drug protection and its sustained-release, microcapsules made of the proteins, bovine- and human serum albumin (BSA and has), encapsulating various hydrophilic and hydrophobic drugs have been developed using various synthesis techniques, including sonochemical synthesis. One such work, by Shimanovich et al., involved the sonochemical encapsulation of ribonucleic acid (RNA) molecules in the BSA microspheres, having an organic core, to study the possibility of using protein microspheres for delivering RNA to Trypanosoma brucei parasites (causes sleeping sickness) and mammalian cells (human U2OS cancer cells). The aim of encapsulation in the microspheres was to protect the RNA molecules from the outer cellular environment and enable their controlled release from the microspheres [16]. Various organic solvents, such as dodecane, soya bean oil, canola oil, and olive oil, were tested to form the organic core of the microspheres, among which the soya bean oil was found to be the most biocompatible to the Trypanosoma brucei parasites and U2OS cancer cells. The RNA molecules were successfully encapsulated inside the BSA microspheres, with 60% encapsulation efficiency, causing no damage to the RNA molecules during encapsulation. The RNA molecules were initially found to be localized in the hydrophobic organic core of the microspheres but delocalized themselves into the hydrophilic BSA crust within ca. 24 h after the formation of the spheres. The average size of the RNA-loaded BSA microspheres (RNA@BSAMS) ranged from 0.5 μm to 2.5 μm, which depended on the size of the encapsulated RNA molecules, whereas the surface charge on the RNA-BSAMS was around −40 meV. RNA@BSAMS were observed to degrade slowly over five months (at 4 °C), while gradually releasing the RNA molecules, thus ensuring the slow and controlled release of RNA by the BSA microspheres. A successful in vitro uptake of RNA@BSAMS by Trypanosoma brucei parasites and U2OS cancer cells was observed spontaneously without the help of additional mediators. The RNA@BSAMS were stable in the cellular environment of the two types of cells, thus proving that RNA remained protected inside the BSA microspheres. 

In the follow-up work, Shimanovich et al. functionalized RNA-loaded BSA microspheres by coating their surface with polymers, either polyvinyl alcohol (PVA) or polyethyleneimine (PEI), to enable targeted delivery of the RNA to the cell nucleus of Trypanosoma brucei parasites and U2OS cancer cells [35]. They observed changes in the surface charge from −40 meV to 0 meV upon coating with PVA and to +20 meV upon coating with PEI. The enhanced cell uptake of the coated microsphere, which was four times larger than the uncoated microspheres, was attributed to the changes in the surface charge. Moreover, unlike the uncoated microspheres, which were localized near the cell membrane, the microspheres coated with PVA localized themselves near the cell nucleus, and the ones coated with PEI were able to penetrate the cell nucleus, thus enabling targeted delivery into it. In another study, Grinberg et al. demonstrated significant inhibition of pancreatic cancer cells (AsPC1) proliferation using antibody-modified BSA microcapsules, loaded with the FDA-approved anti-cancer drug gemcitabine [20]. It is known that the pancreatic cells have an overexpression of EGFR (epidermal growth factor receptor), and one of the strategies of inhibiting their growth is to inhibit the EGFR signaling pathway. For implementing this strategy, anti-EGFR-modified BSA microcapsules, loaded with gemcitabine (BSA-Gem-EGFR), were synthesized sonochemically. The core of the microcapsules was made of dodecane, and gemcitabine was found embedded in the BSA shell matrix. The average size of the obtained antibody-modified microcapsules was ~1.1 μm with the maximum 30% loading capacity for gemcitabine. They demonstrated that BSA microcapsules alone, when incubated with AsPC1 cells, do not show any inhibition in cell proliferation. However, BSA-Gem-EGFR displayed significant inhibition of proliferation of AsPC-1 cells (up to 31%), as compared to controlled gemcitabine-free (cell inhibition up to 15%) and unmodified gemcitabine-loaded BSA microspheres (cell inhibition up to 25%). The strategy used in that work may be effective for treating cancer cells exhibiting an overexpression of EGFR. 

Qian et al. prepared protein-lipid nanocapsules of fluorescently tagged-BSA (FITC-BSA) shells with double emulsion features, wherein oily shell containing PLGA-linolic acid encapsulated a protein-containing aqueous core for the co-delivery of lipophilic paclitaxel and hydrophilic transcription factor p53 for cancer theragnostic [25]. Prepared by the double emulsion technique, the obtained nanocapsules were ~180 nm in diameter, with a zeta potential of −36.4 mV. Paclitaxel was loaded within the oily shell, containing PLGA and linolic acid, whereas p53 resided in the aqueous core of the nanocapsules. Paclitaxel and p53 synergistically induced ca. 100% apoptosis in the HeLa cells, significantly higher than either paclitaxel or p53 alone. The BSA-FITC shell of the nanocapsules could enable the observation of apoptotic cells under a fluorescence microscope. Such a formulation with therapeutic and diagnostic ability has excellent potential in biomedical applications.

Organic core HSA-shell capsules have also been prepared. Gaber et al. developed HSA-based nanocapsules with an oily core, containing a combination of hydrophobic drugs, exemestane, and hesperetin for the targeted breast cancer therapy [10]. A two-stage polymer coating method was applied to make the HSA nanocapsules, wherein an oil-in-water emulsion, containing exemestane in the oil phase and hesperetin added later to the oil phase, was prepared to form a cationic nanoemulsion. The negatively charged HSA shell was deposited on the oily core by adding the aqueous solution of HSA dropwise to the cationic nanoemulsion under stirring. 3-Aminophenylboronic acid (APBA) conjugated HSA was used by this method to prepare functionalized nanocapsules containing exemestane and hesperetin. The HSA nanocapsules and APBA–HSA nanocapsules were both obtained, in the size of ca. 172, which is suitable for delivery into cancer cells. The average zeta potentials were 20.7 ± 1.3 mV and 16.5 ± 2.8 mV, respectively. In-vitro drug release studies showed a biphasic release profile, indicating a diffusion-controlled system. Increased cell internalization of drug-loaded HSA and APBA–HSA capsules was observed in MCF-7 cell lines, compared to free drugs. The ABPA–HSA nanocapsules were successfully able to passively target the hypervascular breast tumor and actively target the overexpressed receptors in this tissue. In vivo studies showed a significant reduction in tumor volume, decreased cell proliferation, and accelerated necrosis when drug-loaded APBA–HSA nanocapsules were introduced. The study successfully utilized the superior synergistic effect of the two hydrophobic drugs, exemestane, and hesperetin, by their targeted delivery into the tumor cells using APBA functionalized HSA nanocapsules. Various other studies have also demonstrated the successful targeted delivery of drug-loaded HSA capsules by functionalizing HSA crust using various biomolecules. Rollet et al. prepared folic acid (FA) functionalized HSA nanocapsules to demonstrate cell-specific internalization by folate receptor (FRβ) macrophages, which are known to be expressed by chronically activated macrophages responsible for inflammation and tissue degradation in Rheumatoid Arthritis patients [22]. The HSA capsules (having an organic core of dodecane) were prepared sonochemically and functionalized with folic acid post-synthesis. The obtained FA-HSA nanocapsules of size ~440 nm and surface charge around −20 mV were able to successfully internalize in positive FRβ macrophages. It was observed that the FA modified HSA nanocapsules were taken up three-fold higher in concentration by FRβ-positive macrophages than in macrophages not expressing FRβ, thus paving the way for the targeted delivery into inflammation-causing macrophages during Rheumatoid Arthritis. In a recent study by Skoll et al., wheat germ agglutinin-functionalized HSA nanocapsules, with a core composed of biocompatible plant oils, were sonochemically prepared for the targeted delivery into urothelial cancer cells [105]. Various oils such as almond oil, rapeseed oil, olive oil, and linseed oil were incorporated in the HSA nanocapsules to form the organic core and analyzed for their effect on the size and stability of the nanocapsules. HSA nanocapsules with olive oil core, obtained in size range of 830–900 nm, proved to possess long-term stability (in the suspension and after freezing). Studies on Human urothelial-5637 cell lines indicated a significantly higher uptake of wheat germ agglutinin functionalized HSA nanocapsules as compared to the unfunctionalized HSA capsules. 

### 4.2. Spherical Protein Capsules with a Solid Core

Protein capsules of hydrophobic and hydrophilic solid cores have been developed to encapsulate hydrophobic and hydrophilic payload. As mentioned in Section 2, hydrophobic payloads have usually been encapsulated to enhance their bioavailability by altering their water solubility. Parthasarathi et al. prepared vitamin E ((+)-α-tocopherol)-loaded whey protein isolate (WPI) microcapsules, altering the solubility of vitamin E, using a combination of spray drying and freeze-drying techniques, which they designated as spray freeze-drying method [12]. The microcapsules consisted of an organic core of solidified sunflower oil containing vitamin E. The synthesis methodology involved firstly the formation of a nanoemulsion of vitamin E and sunflower oil (in an aqueous solution of a surfactant) using a microfluidizer, which was then homogenized with the shell material, WPI, where the WPI molecules were rapidly absorbed on the emulsion interface to form a continuous shell layer around the oil droplets. The homogenized mixture of the organic nanoemulsion and the shell material was then spray-dried to form solid microcapsules with an average size of 145.3 µm, which were later freeze-dried. Vitamin E was encapsulated inside the microcapsules with 89.3% efficiency and seemed to be localized in the core matrix formed of solidified sunflower oil. In-vivo testing on male Wister rats, facilitated by orally administering vitamin E-loaded WPI microcapsules, revealed better pharmacokinetics, with an almost two-fold increase in the oral bioavailability of vitamin E. Increase in the bioavailability of water-insoluble anti-fungal agents has also been observed, due to their encapsulation by microcapsules. For this purpose, Li et al. developed itraconazole-loaded gelatin microcapsules using the spray-drying technique, during which itraconazole was dissolved in a mixture of dichloromethane and ethanol, which was added to an aqueous solution of gelatin and citric acid. This solution was then spray dried to give gelatin microcapsules, containing a solid core of citric acid-containing itraconazole [21]. During the preparation, itraconazole changed from the insoluble crystalline form to the soluble amorphous form, which was an important factor, apart from its acidic microenvironment, due to the presence of citric acid, contributing to a 10-fold enhancement in the solubility of the drug. In-vivo studies on 6–9 week old male Sprague–Dawley rats revealed higher concentrations of the drug in the blood plasma after the oral administration of itraconazole-loaded gelatin microcapsules, compared to the commercial product. 

Microcapsules of protein shells containing hydrophilic polymeric solid core have been developed for the protection of various hydrophilic drugs, as well as probiotic cells. In a study by Laelorspoen et al., alginate microspheric core containing Lactobacillus acidophilus, a probiotic bacteria, were synthesized using the electro-spraying technique and then coated with citric acid-modified zein protein shell layer [11]. The rationale behind zein coating was to protect probiotic cells contained within the matrix of the solid alginate protein core from the harsh gastric environment, given that zein, being highly hydrophobic, possesses good resistivity against degradation due to gastric acids. The sizes of the obtained microcapsules depended upon the concentration of citric acid and ranged from 543 to 650 µm. The effects of electro-spraying voltage and citric acid concentration on the viability of encapsulated probiotic cells were evaluated. It was observed that an increase in the electro-spraying voltage reduced the size of the obtained microcapsules, which, in turn, adversely influenced the survival number of the encapsulated probiotic cells. Moreover, an increase in the citric acid concentration, during the zein protein coating, resulted in a pH decrease in the microenvironment of the cells within the capsules, reducing the number of viable probiotic cells. This is because probiotic cells cannot survive at a pH lower than 2. In-vitro studies in simulated gastric conditions (pH 1.2) revealed a five-fold increase in the cell survival number of the probiotic cells encapsulated within alginate-zein core–shell microcapsules, compared to free probiotic cells. Moreover, zein coating over alginate core proved to be highly effective in protecting the probiotic cells, establishing its supremacy over previously reported [132], uncoated probiotic cell-alginate microspheres. 

Along with their application as drug delivery carriers, protein-based microcapsules with a solid core of other natural polymers have been developed for their use as embolizing agents in cancer therapy, due to their biodegradable and biocompatible nature. In a recent work by Chen et al., adriamycin hydrochloride-loaded sodium alginate-core encapsulated within silk fibroin protein-shell microcapsules were prepared as transcatheter arterial chemoembolizing agents for the treatment of hepatocellular carcinoma, using the emulsified cross-linking method [14]. The method involved emulsifying the aqueous sodium alginate and silk fibroin solution, followed by gelling and cross-linking, facilitated by lowering the pH to 3.5 and adding the cross-linker glutaraldehyde. A stable sodium alginate sphere was formed during the process, due to the acetal reaction, initiated by the chemical interaction of glutaraldehyde with the carboxyl and hydroxyl groups of sodium alginate. Silk fibroin molecules were then deposited on the outer surface of the alginate spheres via iconic and hydrogen bonding. The average size of 142 μm silk fibroin microcapsules were obtained, suitable to act as embolizing agents. The microcapsules showed good blood compatibility and almost no cytotoxicity towards vascular endothelial cells, thus proving safe for intravenous administration of microcapsules. The degradability rate of the microcapsules was found to reach 20.8% in three weeks. It indicated a good degradability trend for the slow release of a chemotherapy drug during the embolization and recanalization of blood vessels. The release mechanism of adriamycin hydrochloride was swelling-controlled, characterized by rapid initial release-kinetics, due to the initial uptake of water by the microcapsules leading to a rapid dissolution of the drug. However, the release kinetics eventually slowed down, due to microcapsule swelling after the water intake. In-vivo embolization studies on the rat ear model revealed that the microcapsules could embolize the arteries in 3 weeks, leading to ischemic necrosis in rats’ ears. Their study, thus, showed that sodium alginate–silk fibroin core–shell microcapsules could effectively serve two purposes: vascular embolization and sustained-drug release, proving their potential as effective embolizing agents. 

Silk fibroin microcapsules have been utilized to encapsulate solid drug-loaded synthetic polymeric-spheres, to enable sustained-release of various drugs. Qiao et al. prepared silk fibroin shell-PLGA-core microcapsules to study the sustained-release of simvastatin (SIM), a cholesterol-reducing drug known for its osteoinductive properties to enable alveolar ridge preservation after tooth extraction [24]. The hydrophobic PLGA core was made porous with a dimpled surface to improve its deposition onto the affected area. Silk-fibroin coating was performed to reduce the initial burst-release of SIM from the microcapsules, as observed in conventional uncoated PLGA microspheres. The preparation process involved synthesizing porous PLGA microspheres, using the emulsion/solvent evaporation technique, wherein an organic solution of SIM-PLGA was emulsified in an aqueous solution, followed by solvent evaporation. The maximum encapsulation efficiency of SIM in the PLGA core was found to be 85%, directly related to the concentration of PLGA. Silk fibroin coating on the PLGA microspheres involved the incubation of PLGA microspheres in an aqueous solution of silk fibroin, followed by glutaraldehyde cross-linking of silk fibroin. In-vitro SIM release studies revealed that initial burst release was reduced to 13.2% in silk fibroin shell-coated PLGA microspheres, compared to the uncoated PLGA microspheres.

### 4.3. Porous/Hollow Core Protein Capsules

The past decade has seen a dramatic rise in systematic studies concerning the utilization of collagen-based microspheres in stem cell therapy, proving their great potential in tissue regeneration applications. Collagen-PLGA hollow core microcapsules functionalized by MnO_2_ nanoparticles (PLGA-Col-MnO_2_) were prepared by Tapeinos et al. to act as scavengers of overexpressed reactive oxygen species (ROS) such as hydrogen peroxide (responsible for oxidative stress in cells, which leads to damaged cellular protein) lipids, membranes and DNA [18]. PLGA hollow core microspheres were prepared by emulsification, followed by collagen-shell coating and incorporation of MnO_2_ nanoparticles (~15 nm). It was shown that MnO_2_ is capable of completely decomposing H_2_O_2_ into water and oxygen without forming an intermediate hydroxyl radical while turning itself into the easily excretable Mn^+2^ ions. In this study, the embedding of MnO_2_ nanoparticles in the collagen-coating of the PLGA microspheres ensured their stability, facilitated better circulation in the bloodstream, prevented their easy removal by macrophages, and preserved their ability to scavenge H_2_O_2_ to release oxygen. In-vitro studies on the two oxidative stress-induced immortalized cell lines, 3T3 and MCF7, revealed that the microspheres could prevent H_2_O_2_-induced cell apoptosis by scavenging on H_2_O_2_ and releasing oxygen, which was cell-specific and was directly affected by PLGA-Col-MnO_2_ microsphere concentration.

Collagen has been transformed into capsules and spheres to deliver substances, such as drugs, growth factors, progenitor cells, etc., for bone cancer therapy, tissue engineering, and bone regeneration applications. Nagai et al. synthesized injectable collagen microspheres loaded with recombinant human vascular endothelial growth factor (rhVEGF) aiming to protect rhVEGF from early degradation in the body and demonstrate its sustained-release to promote angiogenesis [28]. The collagen microspheres were synthesized using the emulsification technique and impregnated with rhVEGF post-synthesis. Collagen microspheres of sizes 1–30 µm possessing a positive surface charge of 8.86 mV (in phosphate-buffer saline) and 3.15 mV (in the culture medium) were obtained. Sustained-release of rhVEGF from the collagen microspheres was observed over four weeks due to the slow degradation of the microspheres. The released rhVEGF maintained its bioactivity and was able to induce capillary formation in human umbilical vein endothelial cells. In another study by Yang et al., collagen microspheres loaded (during synthesis) with steroidal saponins, a glycoside with osteoinductive properties, were synthesized using the emulsion/solvent evaporation technique [133]. They aimed to demonstrate the sustained-release of steroidal saponins from the collagen microspheres and evaluate the osteogenic properties of the composed formulation. The release of steroidal saponins from collagen microspheres was erosion-controlled, facilitated by the degradation of the microspheres in the PBS buffer. In-vitro release studies on pre-osteoblastic MC3T3-E1 cells revealed an increased and sustained expression of alkaline phosphatase (ALP), an enzyme that induces the formation of osteoblasts for bone regeneration. Liu et al. used collagen microspheres as carriers for the sustained-release of basic Fibroblast Growth Factor (bFGF) [31]. The collagen microspheres were cross-linked using different concentrations of carbodiimide to avoid their fast biodegradation and initial burst release of bFGF. The average diameter of the resultant microspheres ranged from 600–3000 nm. The authors observed a significant increase in the stability of bFGF-loaded collagen microspheres, which reduced initial burst release. Sustained-release of bFGF has also been demonstrated by porous silk fibroin (SF) microspheres prepared by Qu et al., in the size range 95–260 µm and the pore size of 1.5–7.0 µm using high voltage electrostatic differentiation, followed by lyophilization [39]. They observed a sustained biphasic release of bFGF from the porous SF microspheres when bFGF was loaded into the microspheres during synthesis, compared to its absorption into the microspheres post-synthesis. Moreover, the culture of mouse embryonic lung fibroblast cells L929 on the bFGF loaded SF microspheres exhibited significant cell proliferation in 5–9 days with very high cell viability and number compared to their culture on bFGF unloaded SF microspheres. 

Hollow/porous microcapsules/spheres have also been studied as scaffolds for growing stem cells and facilitating progenitor cell delivery to a variety of damaged tissues for their regeneration. Such a need arises because stem cells introduced directly to the lesion-affected tissue do not survive long enough to undergo cell differentiation and promote tissue regeneration. For this purpose, Yao et al. utilized collagen microspheres for culturing oligodendrocyte progenitor cells (OPC) to study if the microspheres support cell progenitor growth and differentiation [13]. The collagen microspheres, synthesized using the water-in-oil emulsion technique, were obtained in sizes ranging from 73–192 µm and could support the growth and differentiation of OPC (derived from 2 rats) into oligodendrocytes. When co-cultured with dorsal root ganglion (taken from a 15-day old rat embryo), the oligodendrocytes grown from OPC-collagen microspheres could form neurite myelin sheath and initiate other processes in the dorsal root ganglion. In a series of studies by Chan et al., various types of cells, such as mesenchymal stem cell (MSC), mesenchymal stromal cells, osteoarthritis chondrocytes, and neuroblastoma cells, were microencapsulated in collagen microspheres to study their survival, growth, and differentiation, along with the potential usage of collagen microspheres as in-vitro 3D culture platforms [27,134,135]. In one of those studies, two different sets of MSC-loaded collagen microspheres (MSC@CM), one with undifferentiated MSC@CM and the other with differentiated MSC@CM, to check the effect of cell density and differentiation on cartilage repair [27]. It was observed that undifferentiated MSC@CM implanted at the affected area led to the formation of thicker but softer cartilage, whereas differentiated MSC@CM promoted the growth of stiffer but thinner cartilage. Additionally, the introduction of higher cell density into the affected area favors cartilage regeneration. In another series of studies, Cardier et al. introduced bone marrow MSC@CM into the platelet-rich blood (PRB) clots (MSC@CM-PRB), to induce bone regeneration in non-union lesions and fractures. New bone formation could be observed at the nonunion fracture areas, after three to five months of implanting MSC@CM-PRB into three patients (aged 27, 43, and 81). Moreover, no signs of in-situ abnormalities were observed. The patients’ non-union fractures were healed entirely after 14 months to three years, restoring full functionality and the ability to walk [136,137]. 

Similar to collagen, gelatin microcapsules and silk fibroin microspheres have also been transformed to serve as cell and tissue delivery scaffolds. In a recent work by Hou et al., hyaluronic acid-graft-amphiphilic gelatin hollow microcapsules (HA-AGMC), with shell-embedded superparamagnetic iron oxide nanoparticles (SPIO nps), were prepared to serve as chondrocytes 3D-culture platforms, to form cartilage tissue-mimicking pellets for the correction of articular cartilage damage [23]. The synthesis first involved the grafting of hyaluronic acid (HA) onto the amino groups of amphiphilic gelatin (AG) and the formation of SPIO nanoparticles. The double emulsion technique was employed to prepare the microcapsules, wherein the aqueous solution of HA-g-AG was added to SPIO chloroform solution to form (w/o) single emulsion, followed by the addition of HA-g-AG aqueous solution to form w/o/w double emulsion, then solvent evaporation, dialysis, and freeze-drying. The microcapsules produced had a hydrophilic hollow core, encapsulated by a hydrophobic bilayer, composed of the amphiphilic gelatin. The hydrophobic SPIO nps resided in the shell matrix, with a loading efficiency of 92.2%, and HA covered the inner and the outer surface of the microcapsules. The microcapsules formed were highly biocompatible to the chondrocytes. HA served to connect the microcapsules to the chondrocytes because of the presence of its receptors (CD44) on the chondrocyte membrane. It was revealed that the HA-AGMC concentration of 170 µg/mL, incubated with chondrocytes for 14 days, resulted in the formation of the largest tissue pellet size of 200 µm, with the attachment efficiency of 90%, higher cell density, and viability in the cartilage tissue-mimetic, as well as a stronger connection of the microcapsules to the chondrocyte extracellular-membrane, as compared to the control groups. Due to the presence of SPIO in the HA-AGMC, the pallets could be subjected to biophysical stimulation (via static magnetic field (MF) and magnet derived sheer stress (S)) for the gene expression of Aggrecan, type I and II collagen, and SOX9, which are essential regulators of chondrogenesis and chondrocyte promotion. It was observed that their expression was dramatically up-regulated when the MF and S were applied to HA-AGMC, containing cartilage tissue pallets. Four weeks of implantation of HA-AGMC cartilage tissue-mimicking pellets in the osteoarthritic male New Zealand rabbits revealed improved retention, biofunctionality, better growth, and ordering of chondrocytes. The presence of SPIO-loaded HA-AGMC in the cartilage tissue-mimicking pallet, as well as the application of the magnetic field, exhibits better growth and ordering of chondrocytes in the pallet, and similar strategies can be applied for tissue repair in a variety of conditions/disease states. In another study, Fang et al. prepared strontium-loaded silk fibroin porous (pore diameter ~ 25 µm) microcarriers as the potential osteoinductive platforms to enable the sustained-release of osteogenesis-promoting strontium ions and the attachment, proliferation, and differentiation of mesenchymal stem cells (BMSCs) [26]. These porous microcarriers were prepared using the w/o emulsion-phase separation technique, followed by freeze-drying and strontium mineralization. They allowed the controlled release of strontium ions and attachment, proliferation, and differentiation of seeded BMCs, which was in contrast with unloaded SF microspheres, due to the limited osteoinductive property of SF. Similar to collagen and gelatin, SF-based microspheres can, thus, be used as potential osteoinductive scaffolds for stem cell growth and differentiation, to prepare injectable tissue engineering vehicles.

### 4.4. Multiwalled Core–Shell Protein Capsules

Zheng et al. modified the conventional LBL technique to successfully fabricate hollow SF nanocapsules with efficient encapsulation of doxorubicin (a cationic antitumor drug), as well as chlorin e6 (an anionic photosensitizer) drugs, with efficiencies of 80% and 90%, respectively, to study their sustained-release [8]. A sacrificial core of polystyrene (~250 nm) was used as a stencil to create the hollow SF nanocapsules. Alternating layers of positively charged aminopropyl triethoxysilane (APTES) were introduced in between the SF layers to promote the growth of negatively charged SF layers. SF nanocapsules with positive or negative surface charge were obtained depending upon whether the last SF layer of the capsules was subjected to APTES treatment and if the loading of chlorin e6 (Ce6) or doxorubicin (DOX), respectively, was required. The encapsulation efficiency of DOX and Ce6 reached 80% and 90%, respectively, as the layers of SF were increased, thus proving that their synthesis strategy was efficient in loading cationic and anion drugs. Burst release of DOX and Ce6 was observed from the SF nanocapsules at pH 6.5, but the slow release was evident at pH 7.4. In-vitro cytotoxicity analysis of unloaded SF nanocapsules on L929 cells and MCF-7 cells revealed a 90% higher viability than the control, proving that the nanocapsules were biocompatible. When treated with DOX- or Ce6-loaded SF nanocapsules, MCF-7 breast cancer cell lines experienced higher apoptosis, compared to the free DOX or Ce6. 

Mashoofnia et al. reported multilayered nanocapsules fabrication by the LbL self-assembly of alginate polyanion and BSA polycation for the pH-responsive release of betamethasone disodium phosphate (BSP), a synthetic glucocorticoid with metabolic, immunosuppressive, and anti-inflammatory activity [17]. SiO_2_ was used as a sacrificial core for the deposition of polyelectrolyte layers. They found that efficient complexation between the two polyelectrolytes for their LbL self-assembly was obtained at an alginate/BSA mixing ratio of 1:4 at pH 4. The 7- and 9-times assembly of alginate and BSA layers resulted in nanocapsules of 170 and 188 nm, respectively. The thickness of each layer was found to be ~5–6 nm. The loaded drug was sustainably released at pH 7.4, due to decreased electrostatic interactions between the alginate and the BSA layers. MTT assay analysis of MCF-7 cell lines indicated that the nanocapsules were biocompatible and suitable for drug delivery applications. 

Protein–polyphenol multiwalled microcapsules have been prepared for the encapsulation and protection of hydrophilic and hydrophobic drugs. Polyphenols possess antioxidant properties, which are crucial to protect the encapsulated drugs and prolong their lifetime. Shutava et al. prepared protein –polyphenol microcapsules of alternative gelatin–epigallocatechin gallate (EGCG) layers, using the LbL technique [36], wherein EGCG polyphenol was used for its anti-cancer and antioxidant activity. It was found that the interaction between the gelatin and EGCG layers was predominantly hydrophobic, and the total EGCG content was up to 30% w/w. In another study, Lomova et al. used the LbL technique to prepare multilayered capsules of BSA protein and polyphenol Tannic acid (TA) to load hydrophilic model drug, tetramethylrhodamine-isothiocyanate labeled BSA (TRITC-BSA) and hydrophobic model drug, 3,4,9,10-tetra-(hectoxy-carbonyl)-perylene (THCP) [33]. For encapsulating the hydrophilic TRITC-BSA, a sacrificial TRITC-BSA loaded-CaCO_3_ microparticle core was first coated with poly-L-arginine hydrochloride (PARG), followed by six bilayers of TA/BSA, to give a core/shell particle. PARG was used to provide a stronger interaction between the CaCO_3_ core and shell TA/BSA layers. TRITC-BSA resided as a solid core in the microcapsules. To load the hydrophobic THCP into the microcapsules, THCP was dissolved in sunflower oil and emulsified with the aqueous solution of TRITC-BSA, wherein the latter stabilized on the THCP containing oil droplets, followed by the absorption of TA and BSA layers. THCP was encapsulated as an oily core in the microcapsules. Enzyme-catalyzed degradation of the capsules was employed using α-chymotrypsin, which enabled the sustained-release of the encapsulated model drugs (Table 5 and Table 6).

**Table 5:** Recent advances in the biomedical applications of protein-based solid/liquid/hollow capsules.

Protein	Shell Composition	Core Type	Shell-Bound API	Core API	Biomedical Function	Type of Therapy	Ref.
Albumins	BSA	Liquid, organic (soybean oil)	RNA	-	Controlled release of RNA and its protection from the outer cellular environment	Gene expression and function	[16]
	PVA and PEI functionalized-BSA protein	Liquid, organic (soybean oil)	RNA	-	Targeted delivery of RNA to the cell nucleus, controlled release, and protection from the outer cellular environment	Gene expression and function	[35]
	Anti-EGFR-modified BSA	Liquid, organic (dodecane)	Gemcitabine	-	Sustained-release of Gemcitabine and EGFR blocking	Pancreatic-cancer therapy	[20]
	FITC-BSA bound liquid organic shell filled with PLGA-linolic acid	Liquid Aqueous	Paclitaxel	Transcription factor p53	Sustained-release synergistic apoptotic effect of hydrophilic and hydrophobic drugs on HeLa cells	Cancer theragnostic	[25]
	Multiwalled, BSA polycation–alginate polyanion layered alternatively	Hollow	Betamethasone disodium phosphate (BSP)	-	Sustained-release of BSP having metabolic, immunosuppressive, and anti-inflammatory activity	Rheumatoid arthritis, Crohn’s disease, etc.	[17]
	Multiwalled, BSA-Tannic acid layered alternatively	Solid, hydrophilic tetramethylrhodamine-isothiocyanate labeled BSA (TRITC-BSA)	-	TRITC-BSA	-	-	[33]
	Multiwalled, BSA-Tannic acid layered alternatively	Liquid, organic (sunflower oil)	TRITC-BSA	3,4,9,10-tetra-(hectoxy-carbonyl)-perylene (THCP)	Co-encapsulation of hydrophobic and hydrophilic drugs for sustained-release and their protection by polyphenol Tannic Acid	All types of therapies	[33]
	3-aminophenylboronic acid functionalized-HSA	Liquid, organic	-	Exemestane and Hesperetin	Cell-specific internalization and Targeted delivery into MCF-7 cell lines and sustained-release	Breast-cancer therapy	[10]
	Folic acid-functionalized HSA	Liquid, organic (dodecane)	Folic acid	-	Cell-specific internalization and Targeted delivery into folic-receptor macrophages	Rheumatoid arthritis	[22]
Whey Protein Isolate (WPI)	WPI	Solid Hydrophobic (sunflower oil)	-	vitamin E ((+)-α-tocopherol)	Enhanced bioavailability of water-insoluble vitamin E	Nutritional therapy	[12]
Collagen	MnO_2_ functionalized-collagen-PLGA	Hollow	-	-	Prevention of oxidative stress-induced protein-, lipid- or DNA damage and cell apoptosis	Cancer therapy, cardiovascular and neurological disorders treatment	[18]
Silk Fibroin	Silk fibroin protein	Solid Hydrophilic (alginate)	Adriamycin hydrochloride	-	Transcatheter arterial chemoembolizing by the microcapsules and controlled release of adriamycin hydrochloride	Liver cancer therapy	[14]
	Silk fibroin protein	Solid Hydrophobic (PLGA)	-	Simvastatin	sustained-release of cholesterol-reducing and osteoinductive simvastatin	Bone regeneration	[24]
	Multiwalled, silk fibroin-APTES layered alternatively	Hollow	chlorin e6 (Ce6) and doxorubicin (DOX)	-	Sustained-release of anti-tumor drug DOX and photosensitizer Ce6	Chemophototherapy	[8]
Zein	Citric acid-modified zein	Solid Hydrophilic (alginate)	-	Lactobacillus acidophilus	Protection of probiotic L. acidophilus from the gastric environment	Nutritional therapy	[11]
Gelatin	Gelatin	Solid hydrophilic (citric acid)	-	Itraconazole	Enhanced bioavailability of water-insoluble itraconazole	Treatment of mycotic infections	[21]
	Hyaluronic acid-graft gelatin hydrophobic shell embedding SPIO	Hollow (hydrophilic)	-	-	Chondrocyte cells 3D-culture platforms to form cartilage tissue-mimicking pellets, magnetic field, and magnetic stress-induced gene expression	Tissue repair (correction of articular cartilage damage)	[23]
	Multiwalled gelatin–epigallocatechin gallate (EGCG) LbL	Hollow	-	-	EGCG layers introduce antioxidant properties to the microcapsules to prolong the lifetime and enhance the effectiveness of encapsulated APIs	Cancer therapy and more	[36]

**Table 6:** Recent advances in the biomedical applications of porous protein microspheres.

Protein	Composition	Biomedical Cargo	Biomedical Function	Type of Therapy	Ref.
Collagen	Collagen microspheres	Recombinant human vascular endothelial growth factor (rhVEGF)	Sustained-release of signal protein rhVEGF	Cardiac muscle repair	[28]
		Steroidal saponins	Sustained-release of Steroidal saponins	Osteogenesis and bone regeneration	[133]
		Oligodendrocyte progenitor cells (OPC)	Culturing OPC and their delivery to lesion-affected tissue for the repair of the neurite myelin sheath	Tissue regeneration	[13]
		Mesenchymal stem cells, mesenchymal stromal cells, osteoarthritis chondrocytes, and neuroblastoma cells	3D cell culture platform for stem cell culture, differentiation, and delivery	Stem cell therapy	[134,135]
		Bone marrow mesenchymal stromal cells	Integration into platelet-rich blood clots and implantation at the nonunion lesion site	Bone regeneration for nonunion fractures	[136,137]
Silk Fibroin	Porous silk fibroin (SF) microspheres	Basic fibroblast growth factor (bFGF)	Sustained-release of bFGF and lowering of biodegradability	Tissue repair	[39]
	Strontium loaded porous SF microspheres	Strontium and mesenchymal stem cell (MSC)	Sustained-release of osteogenic strontium and the culture of MSC	Bone regeneration	[26]

## 5. Concluding Remarks and Future Perspectives

Natural, polymer-based APC carriers, especially proteins and polysaccharides, have been utilized widely in biomedical applications, mainly due to features such as biodegradability, biocompatibility, functionalization capability, low-immunogenicity, and blood compatibility. Amongst polysaccharides, chitosan (and derivatives), cellulose (and derivatives), and alginate have been the most commonly utilized shell candidates in core–shell capsules, whereas BSA, HSA, collagen, gelatin, silk fibroin, and zein proteins rule the polypeptide family. Keratin, resilin, and gliadin are some of the less explored polypeptides as shell-forming polymers for core–shell capsules. Recent research trends indicate an accelerated rate in the development of APC carriers with various core–shell structural configurations. Compared to non-porous and porous sphere structures, core–shell capsule configurations have been proven superior, as they enable the introduction of multifunctionalities, higher cargo loading capacity, and encapsulation efficiency. A variety of hydrophobic and hydrophilic cargo can be simultaneously encapsulated and entrapped in the single- and multiwalled core–shell capsules. Porous spheres, on the other hand, were proven to be advantageous as platforms for cell culture. The majority of research and development in cell carrier platforms seems to utilize porous microspheres, possibly due to the availability of a larger surface area for culture within the pores, as well as the polymer matrix surface. 

Several approaches and techniques have been utilized to synthesize porous spheres and core–shell capsules. The majority of these techniques have been utilized for many decades and have undergone slight modifications over the years to meet the system-specific needs. It is also evident that these techniques largely utilize templating approach, wherein either solid or emulsion templates are prepared and used to guide the formation of core–shell-type, as well as sphere-type structural configurations. Solid templating is the easiest and most direct approach for synthesizing solid- and hollow-core, as well as single- and multiwalled capsules. There are many reports on utilizing the solid templating approach for solid- and hollow-core–shell capsule synthesis. Liquid-core capsules have also been indirectly prepared using solid templating. However, the number of such studies is relatively low. Emulsion templating is another commonly used method of synthesis, especially for oily core capsules and porous spheres. An important difference between sphere and capsule synthesis using emulsion templating is that the latter generally requires the formation of o/w emulsion, as opposed to w/o emulsion, especially when the polymer is hydrophilic and soluble in water. Ultrasonication-assisted emulsification has also been established as one of the leading capsule synthesis approaches, due to its facile and time-efficient methodology. Several studies have utilized the ultrasonication approach for the synthesis of liquid-core capsules. However, the proportion of studies involving the ultrasonic synthesis of oily-core capsules is higher than that of the aqueous-core capsules. Thus, ultrasonication can be further explored towards the synthesis of aqueous-core capsules.

The stability of polymeric carriers, especially core–shell single- and multiwalled capsules, has been a concern. For their long-term stability, covalent crosslinking has been exploited. However, as mentioned earlier, a balance between covalent and non-covalent interactions must be achieved to ensure capsule stability but must enable stimuli-responsive cargo release. Storage is another concern when it comes to core–shell capsules. Liquid-core and hollow core capsules are highly prone to structural deformations and bursting when dried for storage and characterization as dry powders. Liquid-core capsules can be stored as colloidal solutions, instead, to avoid these issues. However, it must be noted that if the colloidal conditions are not appropriately maintained, the capsules suffer aggregation, which results in the loss of shell functionalities. More efforts are needed in the direction of purification and proper storage. In addition, alternative, less invasive approaches for preparing characterization samples of liquid-core capsules are needed.

## Data Availability

Not applicable.
